# Activating the cellular scavenger: A bioactive hydrogel promotes diabetic wounds via plant exosome-like nanovesicles enhanced macrophage efferocytosis

**DOI:** 10.1016/j.bioactmat.2026.03.039

**Published:** 2026-03-24

**Authors:** Yue-Qi Zhang, Rong Nie, Zi-Yuan Feng, Ming-Hui Fan, Zhi-Xue Shen, Xiu-Zhen Zhang, Ji-Ye Zhang, Yan-Lin Jiang, Qing-Yi Zhang, Kai Huang, Li-Ping Mou, Yan-Ming Chen, Hui-Qi Xie

**Affiliations:** a Department of Orthopedic Surgery and Orthopedic Research Institute, Stem Cell and Tissue Engineering Research Center, Frontiers Science Center for Disease-Related Molecular Network, State Key Laboratory of Biotherapy, West China Hospital, Sichuan University, Chengdu, Sichuan, 610041, China; b Tianfu Jincheng Laboratory, Chengdu, Sichuan, 610093, China

**Keywords:** Grape exosome-like nanovesicles, M2c macrophages, Efferocytosis, Decellularized matrix-modified hydrogel, Diabetic wound repair

## Abstract

The core pathological process of impaired diabetic wound healing is closely associated with macrophage homeostasis imbalance and defective efferocytosis. To address this clinical challenge, this study innovatively developed a synergistic therapeutic system combining grape exosome-like nanovesicles (G-ELNs) and decellularized small intestinal submucosa matrix-modified hydrogel (SM). In vitro experiments demonstrated that G-ELNs effectively induced macrophage polarization toward the M2c phenotype and significantly enhanced efferocytosis efficiency by activating the c-Mer Tyrosine Kinase (MERTK) receptor. The SM hydrogel, with its triple-microporous topological structure and dynamic sustained-release properties, provided a long-term localized delivery platform for G-ELNs. In a diabetic rat full-thickness skin defect model, this system exhibited dual regulatory effects: spatially and temporally targeted delivery of exosomes promoted M2c macrophage polarization during the early inflammatory phase, rapidly clearing apoptotic cell debris through enhanced efferocytosis to block inflammatory cascades and transition the healing process to the proliferative phase, while simultaneously accelerating collagen fiber cross-linking and vascular network maturation in the proliferative phase, ultimately expediting wound closure. This study not only elucidates a novel immunomodulatory mechanism based on natural products but also proposes a clinically transformative strategy for efficient diabetic wound management.

## Highlights

• **Innovative System Design**: SM@G-ELNs integrates grape exosome-like nanovesicles (G-ELNs) with modified hydrogel (SM), leveraging G-ELNs' immunomodulatory functions and hydrogel's sustained release for localized long-term delivery adapted to wound microenvironment.
• **Novel Mechanistic Insight**: First evidence shows G-ELNs activate macrophage MERTK receptor to drive M2c repolarization and enhance efferocytosis, interrupting diabetic wound "inflammation-necrosis" cycle.
• **Translational** **Pathway Advancement**: G-ELNs exploit plant-derived nanovesicles' abundant sources, low cost, and scalable production to bypass stem cell exosomes' culture complexity and translational barriers.

In summary, the SM@G-ELNs system with its well-defined mechanism, high biocompatibility, and clinical accessibility provides an innovative, practical, and translatable strategy for diabetic ulcer treatment, significantly expanding the potential of plant-derived nanovesicles in chronic wound repair.

## Graphical abstract

Scheme 1. **Graphical Abstract. Schematic Illustration of the Synergistic Therapeutic System for Diabetic Wound Repair.** The schematic diagram illustrates the synergistic therapeutic mechanism of G-ELNs and SM in diabetic wound repair. G-ELNs specifically activate the MERTK receptor on macrophages, inducing their polarization toward the reparative M2c phenotype and enhancing efferocytosis to clear apoptotic cell debris and block inflammatory cascades. Meanwhile, SM hydrogel utilizes its three-dimensional porous topological structure and dynamic sustained-release properties to achieve targeted delivery and prolonged release of G-ELNs, while promoting fibroblast/endothelial cell proliferation to fuel wound regeneration. In diabetic wounds, this system exerts dual regulatory effects: phenotypically, it reshapes the anti-inflammatory microenvironment by driving macrophage M2c polarization and secretion of pro-repair factors (e.g., IL-10, TGF-β); functionally, it amplifies efferocytosis through the MERTK signaling pathway, efficiently eliminating apoptotic fragments to disrupt the “inflammation-apoptosis-necrosis” vicious cycle. This coordinated action ultimately promotes ordered collagen fiber crosslinking and vascular network maturation, accelerating tissue regeneration.

**Figure:**
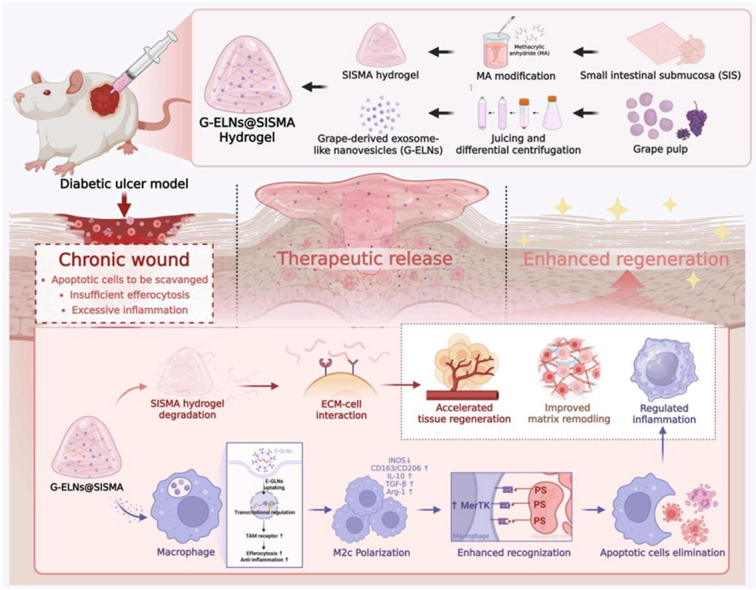

## 1 Introduction

Diabetes mellitus is one of the leading global causes of disability and mortality [1]. Prolonged hyperglycemia in diabetic patients predisposes them to various complications, among which diabetic skin ulcers (DSUs) are one of the most devastating [2,3]. With approximately 537 million diabetic individuals worldwide, the prevalence of DSUs ranges from 19% to 34% across regions and populations. DSUs are characterized by chronic, non-healing wounds often accompanied by persistent inflammation, recurrent infections, and high amputation risks [4,5]. Despite advancements in standard therapies (e.g., debridement, negative pressure wound therapy, skin flap transplantation), over 60% of patients still face challenges of delayed healing and high recurrence rates. Consequently, identifying and developing novel therapeutic strategies for DSUs is critically urgent [[6], [7], [8]].

Existing studies indicate that the fundamental pathological mechanism of such wounds is closely linked to immune microenvironment dysregulation, particularly macrophage dysfunction leading to impaired repair progression [9,10]. The classically activated M1 macrophages persistently dominate the microenvironment, where their excessive secretion of pro-inflammatory cytokines impedes tissue repair. In contrast, through the alternative activation pathway, M2 macrophages play a central regulatory role in inflammation resolution, tissue remodeling, and efferocytosis by secreting anti-inflammatory mediators such as IL-10 and TGF-β. As a fundamental biological mechanism for maintaining tissue homeostasis, efferocytosis—mediated by professional phagocytes (e.g., macrophages) or non-professional phagocytes—specifically recognizes and clears programmed apoptotic cells, serving as a critical “cellular scavenger” in life processes. Its biological significance lies not only in the daily elimination of billions of apoptotic cell debris to sustain tissue renewal and regeneration but, more crucially, in preventing the release of toxic substances (e.g., proteases and reactive oxygen species) from secondary necrosis of uncleared apoptotic cells, thereby fundamentally averting the onset of inflammatory diseases [[11], [12], [13], [14], [15]]. However, under chronic inflammatory conditions in diabetes, the M2 polarization process is severely impaired, leading to a marked decline in efferocytic capacity mediated by macrophages. This results in the accumulation of apoptotic cell debris and the release of damage-associated molecular patterns (DAMPs), thereby establishing a vicious “inflammation-necrosis” cycle that ultimately drives the wound into irreparable healing arrest. Consequently, targeted modulation of macrophage polarization balance to promote their transition to the M2 phenotype and enhance efferocytosis represents a highly promising therapeutic strategy for reversing immune dysregulation in diabetic wounds.

Exosome-like nanovesicles (ELNs), leveraging their natural bioactive component delivery capacity and low immunogenicity, provide a novel tool for modulating the immune microenvironment of diabetic skin ulcers. Previous studies by our research group demonstrated that hypoxia-preconditioned human urine-derived stem cell exosomes effectively promoted diabetic wound healing via miR-486-5p-mediated targeting of SERPINE1, offering critical theoretical foundations for exosome-based repair strategies [16]. However, stem cell-derived exosomes face inherent limitations in clinical translation due to their dependence on complex cell cultures, high costs, and limited yields during large-scale production [[17], [18], [19]]. To address these limitations, plant-derived exosome-like nanovesicles (P-ELNs) have surfaced as a compelling alternative therapeutic strategy [20]. P-ELNs present several distinct advantages: their raw materials are abundant, production costs are low, and yields are high. This profile eliminates the reliance on large-scale cell culture, thereby overcoming the critical yield limitation associated with stem cell-derived exosomes. As naturally occurring nanoscale bilayer vesicles, P-ELNs share structural and functional similarities with their mammalian counterparts, particularly in their capacity to carry bioactive cargo (e.g., proteins, lipids, and nucleic acids) [21]. Furthermore, P-ELNs are characterized by low immunogenicity and high biocompatibility [22]. These inherent properties, combined with the feasibility of scalable production from readily available sources, position P-ELNs as a highly promising platform for meeting clinical application demands [23] [24]. Among various P-ELNs, grape exosome-like nanovesicles (G-ELNs) have garnered significant attention due to their unique composition and functionalities. Enriched with plant-specific miRNAs and polyphenols, G-ELNs can be efficiently internalized by immune cells and exhibit remarkable immunomodulatory and tissue-repairing potential [25,26]. Despite this promise, the application of G-ELNs in the healing of chronic diabetic wounds has not been reported yet, and further exploration is needed. Moreover, the clinical translation of G-ELNs faces a substantial barrier: their susceptibility to enzymatic degradation in the complex wound microenvironment. This instability results in rapid loss of bioactivity and challenges in sustaining effective local concentrations. Hydrogel delivery systems, leveraging their three-dimensional network structure as a physical barrier against wound exudate flushing, enable controlled degradation and sustained exosome release, thereby prolonging the retention of active components and preserving functional stability [[27], [28], [29]]. In our previous work, we developed a decellularized matrix-modified hydrogel (SM), experimentally validated to possess biomechanical strength matching wound tissue, degradation kinetics synchronized with healing progression, and a moisture-retentive microenvironment that synergistically enhances repair, thereby establishing a technical foundation for constructing an efficient delivery platform [16].

**Scheme 1:**
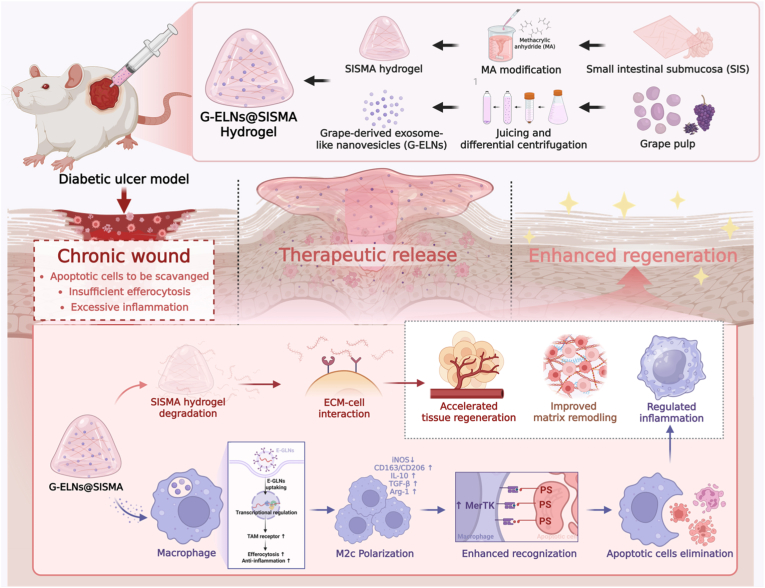
Schematic Illustration of the Synergistic Therapeutic System for Diabetic Wound Repair. The schematic diagram illustrates the synergistic therapeutic mechanism of G-ELNs and SM in diabetic wound repair. G-ELNs specifically activate the MERTK receptor on macrophages, inducing their polarization toward the reparative M2c phenotype and enhancing efferocytosis to clear apoptotic cell debris and block inflammatory cascades. Meanwhile, SM hydrogel utilizes its three-dimensional porous topological structure and dynamic sustained-release properties to achieve targeted delivery and prolonged release of G-ELNs, while promoting fibroblast/endothelial cell proliferation to fuel wound regeneration. In diabetic wounds, this system exerts dual regulatory effects: phenotypically, it reshapes the anti-inflammatory microenvironment by driving macrophage M2c polarization and secretion of pro-repair factors (e.g., IL-10, TGF-β); functionally, it amplifies efferocytosis through the MERTK signaling pathway, efficiently eliminating apoptotic fragments to disrupt the “inflammation-apoptosis-necrosis” vicious cycle. This coordinated action ultimately promotes ordered collagen fiber crosslinking and vascular network maturation, accelerating tissue regeneration.

Therefore, this study aims to construct a synergistic therapeutic system termed “grape exosome-like nanovesicles/decellularized matrix-modified hydrogel” (SM@G-ELNs), systematically elucidating its mechanism in breaking the “inflammation-necrosis” vicious cycle of diabetic wounds by driving macrophage reprogramming toward the M2c phenotype and activating efferocytosis pathways to reverse immune imbalance. The findings not only provide a novel strategy combining translational potential with mechanistic innovation for immunotherapy of diabetic ulcers but also deepen the understanding of P-ELNs’ regulatory roles in immunometabolic diseases and expand their application prospects (see Scheme 1).

## 2 Experimental section

### 2.1 Materials and reagents

The high glucose Dulbecco's Modified Eagle Medium (DMEM), F12·nutrient· mixture, RPMI-1640, fetal bovine serum (FBS) and penicillin-streptomycin dual-antibiotic solution (P/S, 100 U/mL penicillin, 100 μg/mL streptomycin) were purchased from Gibco (USA). Phosphate-buffered saline (PBS) was purchased as a pre-sterilized product from OriGene (USA). When photopolymerization was required, methyl methacrylate (MMA) and the photoinitiator LAP were provided by Shanghai Macklin Biochemical (China), and the light-curing device was provided by Suzhou EFL Precision Instruments (China). Porcine pepsin From Sigma (USA). Fresh porcine small intestine tissues were purchased from a local butcher and were used after being ensured to be free of disease. All other chemicals were analytical grade or cell culture-grade and were used as prepared unless otherwise noted.

### 2.2 Cells and experimental animals

Human umbilical vein endothelial cells (HUVECs), NIH-3T3 fibroblasts, Jurkat leukemia cells, Bone Marrow-Derived Macrophages(BMDM) and RAW264.7 macrophages were purchased from Cell Bank of Chinese Academy of Sciences. Culture conditions: HUVECs in DMEM/F12 (1:1) containing 10% FBS and 1% P/S; NIH-3T3 and RAW264.7 in high glucose DMEM containing 10% FBS and 1% P/S; Jurkat cells in RPMI-1640 containing 10% FBS and 1% P/S. All cells were cultured at 37 °C in a humidified 5% CO_2_ incubator and passaged when reaching 80% to 90% confluence with 0.25% trypsin. SPF-grade male SD rats (8 weeks, Sichuan University Experimental Animal Center) were raised in SPF barrier systems (22 ∼ 25 °C, 12 h light/dark cycle) with free access to food/water. Animal experiments were conducted in accordance with the guidelines issued by the National Institutes of Health and were approved by the Ethics Committee of West China Hospital, Sichuan University (No. 20221201005).

### 2.3 Isolation, purification, and characterization of G-ELNs

Fresh Kyoho grape pulp was homogenized in ice-cold PBS (1:3, w/v). The homogenate was then centrifuged stepwise: first at 300×*g* for 10 min to remove large debris, then the supernatant was collected and centrifuged at 2000×*g* for 15 min, and finally the resulting supernatant was centrifuged at 10,000×*g* for 20 min to pellet finer particles. The supernatant was further subjected to sucrose density gradient ultracentrifugation to obtain G-ELNs. The nanovesicles were resuspended in PBS, counted using a BCA protein quantification assay, and analyzed by NTA and TEM for structure characterization. The instrument parameter is listed in the Supplementary Materials.

### 2.4 PKH26-labeled G-ELNs uptake assay in RAW264.7 macrophages and BMDM

G-ELNs were labeled with PKH26 by brief vortexing and subsequent incubation for 10 min in the dark. Free dye was removed by ultracentrifugation. RAW264.7 cells and BMDM were incubated with labeled G-ELNs, fixed with 4% paraformaldehyde (PFA), permeabilized, blocked with 5% BSA, and stained with FITC-phalloidin (cytoskeleton) and DAPI (nuclei). Images were acquired via structured illumination microscopy. The specific experimental parameter are presented as elaborated in the supplementary materials.

### 2.5 Quantitative real-time polymerase chain reaction (qPCR) for gene expression analysis

RAW264.7 cells (3 × 10^5^/well in 6-well plates) were treated with 100 μg/mL G-ELNs in DMEM for 48 h. Total RNA was extracted using a commercial kit (Promega), and 1 μg RNA was reverse-transcribed into cDNA (PrimeScript RT kit, TaKaRa). qPCR was performed with TB Green Premix Ex *Taq*II (TaKaRa) in a 20 μL system. Gene expression of M1 (*iNOS*), M2 (*Il-4*, *Il-10*, *Arg-1*, *Tgf-β*), and efferocytosis-related markers was analyzed using the 2^–ΔΔCt^ method, normalized to GAPDH. The specific experimental steps are presented as elaborated in the supplementary materials.

### 2.6 EdU staining for RAW264.7 cell proliferation

RAW264.7 cells were seeded in 24-well plates and treated with G-ELNs (0, 10, 100, 1000 μg/mL) for 24 h. Cell proliferation was assessed using an EdU assay kit (Beyotime) according to the manufacturer's instructions, with nuclei counterstained by DAPI. The percentage of EdU-positive cells was quantified from five random fields per well using ImageJ, and the experiment was repeated three times independently. The specific experimental steps are presented as elaborated in the supplementary materials.

### 2.7 Polarization phenotype analysis of RAW264.7 cells

#### 2.7.1 Flow cytometry for M1/M2 phenotypes

We exposed macrophages to G-ELNs under standardized culture conditions and then they were stained with PE-anti-CD86 and APC-anti-CD206 antibodies (BioLegend) to identify M1 and M2 macrophages. The percentages of CD86^+^ and CD206^+^ populations were analyzed by flow cytometry (BD CytoFLEX). You'll find detailed protocols for antibody validation and ratio normalization within the Supplementary Materials.

#### 2.7.2 Confocal laser scanning microscopy for M2 markers

RAW264.7 cells were treated with G-ELNs, then fixed, permeabilized, and incubated with a validated antibody targeting M2-associated surface receptors (CD163 and CD206). Fluorescence signals were acquired using optimized parameters, and the mean fluorescence intensity (MFI) was quantified through automated image analysis. The specific experimental steps are presented as elaborated in the supplementary materials.

### 2.8 Polarization phenotype analysis of primary bone marrow-derived macrophages (BMDMs)

Bone marrow cells were harvested from the femurs and tibias of 6-week-old male C57BL/6 mice. The marrow cavity was flushed with serum-free RPMI 1640 medium, and the resulting cell suspension was passed through a 70 μm cell strainer. After centrifugation, red blood cells were lysed. The cell pellet was resuspended in serum-free RPMI 1640, and cells from one mouse were seeded into one culture plate. Following 2 h of incubation, non-adherent cells were removed by washing. The adherent cells were cultured in RPMI 1640 medium supplemented with 10% heat-inactivated FBS and 20 ng/mL M-CSF to induce differentiation, with medium replaced every 2 days. After 7 days of culture, mature macrophages were obtained and confirmed by F4/80 expression before being used for subsequent experiments. Subsequently, primary macrophages were subjected to immunofluorescence staining for CD163 and MERTK, following the same protocol as described above.

### 2.9 Bioinformatics analysis

The RNA-seq data of treated macrophages were subjected to a standard bioinformatics analysis pipeline, including differential gene expression analysis, functional annotation of efferocytosis related pathways, and protein interaction network modeling. Full methodological details of sequencing parameters and computational algorithms are described in Supplementary Methods.

### 2.10 In vitro efferocytosis assays

#### 2.10.1 Flow cytometry for efferocytosis rate

Macrophage efferocytosis capacity was measured by flow cytometry following co-culture with fluorescently labeled apoptotic Jurkat cells. Apoptotic Jurkat cells, induced by UV irradiation and validated by Annexin V/PI staining, were labeled with PKH26. These cells were then co-cultured with G-ELNs or PBS pretreated RAW264.7 macrophages at a 1:5 ratio for 2 h. Following co-culture, efferocytosis was quantified by flow cytometry as the proportion of F4/80^+^ macrophages that were PKH26^+^. The specific experimental steps are presented as elaborated in the supplementary materials.

#### 2.10.2 Immunofluorescence-based efferocytosis index

Following a 48-h pretreatment with G-ELNs, RAW264.7 macrophages and BMDM were co-cultured with PKH26-labeled apoptotic Jurkat cells (1:5 ratio) for 2 h to assess efferocytosis. After fixation, permeabilization, and staining of the cytoskeleton (FITC-phalloidin) and nuclei (DAPI), high-content confocal microscopy (PerkinElmer) was employed for image acquisition. Comprehensive experimental details are provided in the Supplementary Materials.

### 2.11 Sequential efferocytosis assay via flow cytometry

To assess sequential efferocytosis, RAW264.7 macrophages (pretreated with PBS or G-ELNs for 24 h) were first co-cultured with PKH26-labeled apoptotic Jurkat cells (1:5 ratio) for 2 h. After washing, the same cells were subsequently exposed to a second batch of PKH67-labeled apoptotic targets (1:5 ratio) for an additional 2 h. Macrophages (F4/80^+^) that had engulfed both batches (PKH26^+^, PKH67^+^) were quantified by flow cytometry. Comprehensive experimental details are provided in the Supplementary Materials.

### 2.12 MERTK functional blockade assay

#### 2.12.1 Screening of UNC2025 concentrations

To investigate whether G-ELNs enhance efferocytosis through MERTK receptor activation, a loss-of-function assay was conducted using the specific MERTK inhibitor UNC2025 (MedChemExpress). Briefly, RAW264.7 murine macrophages were treated with varying concentrations of UNC2025 (1, 10, 100, and 500 nM). Cell viability was assessed on days 1, 3, and 5 using the CCK-8 assay to rule out cytotoxic effects. Concurrently, the inhibitory efficacy of UNC2025 on MERTK expression was evaluated by Western Blot analysis. Based on the optimal balance between minimal cytotoxicity and maximal MERTK inhibition, the appropriate concentration of UNC2025 was selected for subsequent experiments.

#### 2.12.2 MERTK blockade confirms its role in G-ELNs-mediated efferocytosis

For the functional blockade assay, BMDMs were divided into three groups: (1) Control group (treated with apoptotic cells only), (2) UNC2025 inhibitor group (pretreated with 10 nM UNC2025 for 4 h, followed by co-incubation with apoptotic cells and G-ELNs), and (3) G-ELNs group (treated with apoptotic cells and G-ELNs without inhibitor). After co-incubation, cells were harvested, and the expression levels of MERTK and ARG1 (a downstream effector of efferocytosis) were detected by Western Blot analysis.

### 2.13 Preparation of small intestinal submucosa (SIS)

SIS was prepared according to our previously established protocol [16,30]. In brief, the SIS was prepared through mechanical delamination, decellularization via sequential lipid/protein removal with organic solvents and enzymatic treatment, and freeze-drying. The resulting SIS was then digested with pepsin to generate a scaffold for subsequent SM preparation. Comprehensive experimental details are provided in the Supplementary Materials.

### 2.14 SM preparation and characterization

Lyophilized SIS was cryo-ground into a fine powder and subsequently digested containing pepsin at 25 °C for 72 h. The solution was functionalized by adding methyl methacrylate (MMA; 1:1 mass ratio to SIS), adjusting the pH to 7.0, and stirring for 24 h to yield methacrylated SIS (SM). After dialysis and lyophilization, SM hydrogel was formed by dissolving the powder in PBS with 1% LAP and photo-crosslinking under 405 nm light. The SM was characterized by SEM. Comprehensive experimental details are provided in the Supplementary Materials.

### 2.15 Biocompatibility evaluation of SM hydrogel

#### 2.15.1 CCK-8 proliferation assay

Cell proliferation upon material exposure was monitored with a standardized colorimetric assay. We conducted absorbance measurements at set intervals to assess metabolic activity. Supplementary Materials present the outlined rules for reagent preparation and normalization.

#### 2.15.2 Live/dead staining

Cells (3 × 10^4^/well in 24-well plates) were treated with extracts for 3 days, stained with calcein-AM (1 μg/mL)/propidium iodide (1 μg/mL), and imaged via confocal microscopy.

#### 2.15.3 Hemolysis assay

The assessment of hemocompatibility was done via standardized erythrocyte lysis protocols. We isolate red blood cells, incubate them with hydrogel extracts under physiological conditions, and quantify hemolysis rates via spectrophotometric analysis. Detailed centrifugation parameters along with normalization criteria are found in Supplementary Materials.

### 2.16 In vitro release profile of G-ELNs from SM hydrogel

We probed the sustained release kinetics of G-ELNs from hydrogels by dynamic incubation in physiological buffer. Cumulative release was measured periodically via colorimetric protein quantification. Supplementary Methods give an outline of full sampling intervals and buffer composition details.

### 2.17 Diabetic full-thickness skin defect model and wound treatment

A diabetic model was induced by STZ in male SD rats. One week after the injection, rats with sustained hyperglycemia (fasting blood glucose ≥ 16.67 mmol/L) were randomly assigned to five groups (n = 4 per group). Four groups received full-thickness skin defects and were treated with: Saline, SM hydrogel, free G-ELNs, and G-ELNs@SM hydrogel. The fifth group, comprising diabetic rats without skin wounds, served as a non-wounded diabetic control to assess the systemic baseline. Supplementary Protocols contain the descriptions of the animal selection criteria and surgical protocols.

### 2.18 Histological and immunohistochemical analysis of wound tissue

At designated time points (days 3, 7, 14, 21), wound tissues were harvested, fixed in formalin, and processed into paraffin sections. Histological evaluation was performed using H&E (for inflammation and granulation tissue) and Masson's trichrome (for collagen deposition). Collagen typing was analyzed via picrosirius red staining under polarized light. For immunohistochemistry, sections were incubated with primary antibodies against CD163, CD68, Caspase-3, and MERTK, followed by corresponding secondary antibodies and DAB development. Comprehensive experimental details are provided in the Supplementary Materials.

### 2.19 Statistical analysis

All data were derived from no fewer than three independent replicates and are expressed as mean ± standard deviation. To assess group differences, one-way ANOVA coupled with Tukey's post hoc analysis was applied GraphPad Prism 10.0. Statistical significance was defined as *∗P < 0.05*, ∗∗*P < 0.01*, and ∗∗∗*P < 0.001*, denoting the comparison with the specified group.

## 3 Results and discussion

### 3.1 Characterization and concentration screening of G-ELNs

**Fig. 1:**
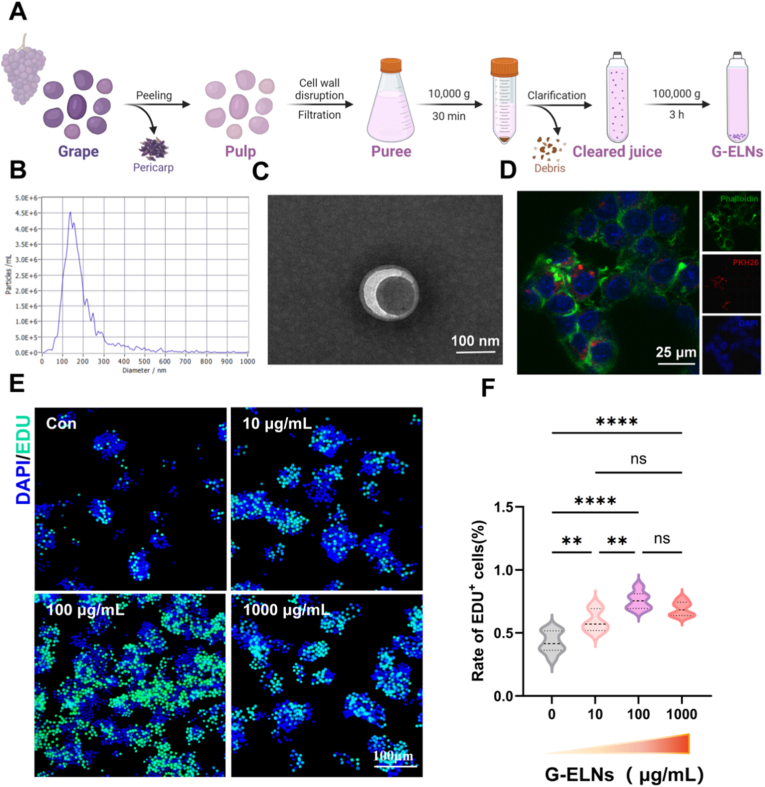
**Characterization and concentration screening of G-ELNs.** (A) Process of exosome extraction from grapes. (B) NTA of particle size distribution for grape-derived exosomes. (C) TEM images of grape exosomes. (D) Confocal microscopy images of RAW264.7 cellular uptake of G-ELNs. (E) Proliferation of RAW264.7 cells detected via EdU labeling assay. (F) Quantitative analysis of proliferation rates. Data from 2 independent experiments (n = 2 experiments), 6 biological replicates per group, 3 technical replicates per sample. *ns:p*>*0.05*; *∗p < 0.1, ∗∗p < 0.01, ∗∗∗p < 0.001, ∗∗∗∗p < 0.0001.*

Exosome-like nanovesicles were extracted from purple grape pulp (PPu@G-ELNs), purple grape peel (PPe@G-ELNs), white grape pulp (WPu@G-ELNs), and white grape peel (WPe@G-ELNs) using differential centrifugation. PPu@G-ELNs exhibited uniform particle size (50 ∼ 120 nm, mean diameter 85.6 ± 12.3 nm) and a high concentration of (4.5 × 10^6^ ± 0.5 × 10^6^) particles/mL, significantly outperforming other sources (e.g., white grape pulp-derived exosomes with particle sizes of 130 ∼ 180 nm and a concentration of 2.1 × 10^6^ particles/mL) (Fig. S1). To assess the purity of the isolated G-ELNs, we performed Western Blot analysis to detect the presence of exosomal markers and a negative control protein. As shown in Fig. S3, we found that G-ELNs were positive for both HSP70 and TET8, two well-established exosomal markers, confirming their vesicular identity. In contrast, β-actin, a cytosolic protein commonly used as a negative control for vesicle contamination, was undetectable in the G-ELN samples. These results demonstrate that the purified G-ELNs are of high purity and free from significant contamination by cellular debris or non-vesicular proteins. Transmission electron microscopy (TEM) further revealed that PPu@G-ELNs exhibited typical cup-shaped/spherical morphology (Fig. 1C) and no aggregation (Fig. S1D–F), while particles from other three groups aggregated (Fig. S1G). qPCR analysis showed that PPu@G-ELNs-treated groups had significantly higher mRNA expression levels of *Il-10*, *Arg-1* and *Tgf-β* compared to other treatment groups (Fig. S2), suggesting that PPu@G-ELNs had the strongest M2 macrophage-polarizing effect. According to the comprehensive physicochemical characterization and functional validation, PPu@G-ELNs were selected as experimental material for the subsequent studies (hereafter uniformly referred to as G-ELNs).

G-ELNs were successfully isolated from grape pulp via differential centrifugation. The workflow, which involves homogenization, filtration, differential centrifugation, and ultracentrifugation purification, is shown in Fig. 1A. Nanoparticle tracking analysis (NTA) showed that G-ELNs exhibited particle size distribution of 50 ∼ 120 nm, mean diameter of 85.6 ± 12.3 nm, and concentration of 4.5 × 10^6^ ± 0.5 × 10^6^ particles/mL (Fig. 1B), which was consistent with the typical size range of exosomes (30 ∼ 150 nm). TEM results showed that G-ELNs had typical morphology of cup-shaped or spherical particles with clear boundaries and no obvious aggregation (Fig. 1C), which further verified the exosome-like property [31,32].

To validate the interaction between G-ELNs and macrophages, we first performed the cellular uptake experiment. PKH26-labeled G-ELNs (red fluorescence) were co-incubated with RAW264.7 cells for 3 h. Confocal microscopy showed that red fluorescence was mainly localized in the cytoplasm (Fig. 1D), which indicated that G-ELNs could be efficiently uptaken by macrophages and provided the prerequisite for further regulation of macrophage functions.

In the EdU proliferation assay, RAW264.7 cells were treated with different concentrations of G-ELNs (10 μg/mL, 100 μg/mL, and 1000 μg/mL). The 100 μg/mL treatment group showed the highest proliferation rate. Therefore, 100 μg/mL was chosen for the subsequent experiments. Adequate population of macrophages is the cellular basis for effective debridement, resolution of inflammation, and initiation of repair in the tissue. Pro-proliferative activity of G-ELNs directly enlarges the “cellular reservoir” of macrophages in the microenvironment of wound.

The above results indicate that G-ELNs can significantly promote the proliferation of macrophages. Macrophages, as the core of the innate immune system, the maintenance and expansion of their cell numbers are the basis for performing immune surveillance, inflammatory regulation, and tissue repair functions [33]. The G-ELNs used in this study, as a natural nanoscale delivery system, their phospholipid bilayer structure can effectively protect the contents and achieve precise delivery of bioactive components to target cells [34]. We speculate that the active components such as lipids, small molecule metabolites, and plant-derived microRNAs contained in G-ELNs may drive the proliferation of macrophages by mimicking or activating the key proliferative signaling pathways within macrophages [35].

### 3.2 G-ELNs promote M2c phenotype polarization in RAW264.7 macrophages *In vitro*

**Fig. 2:**
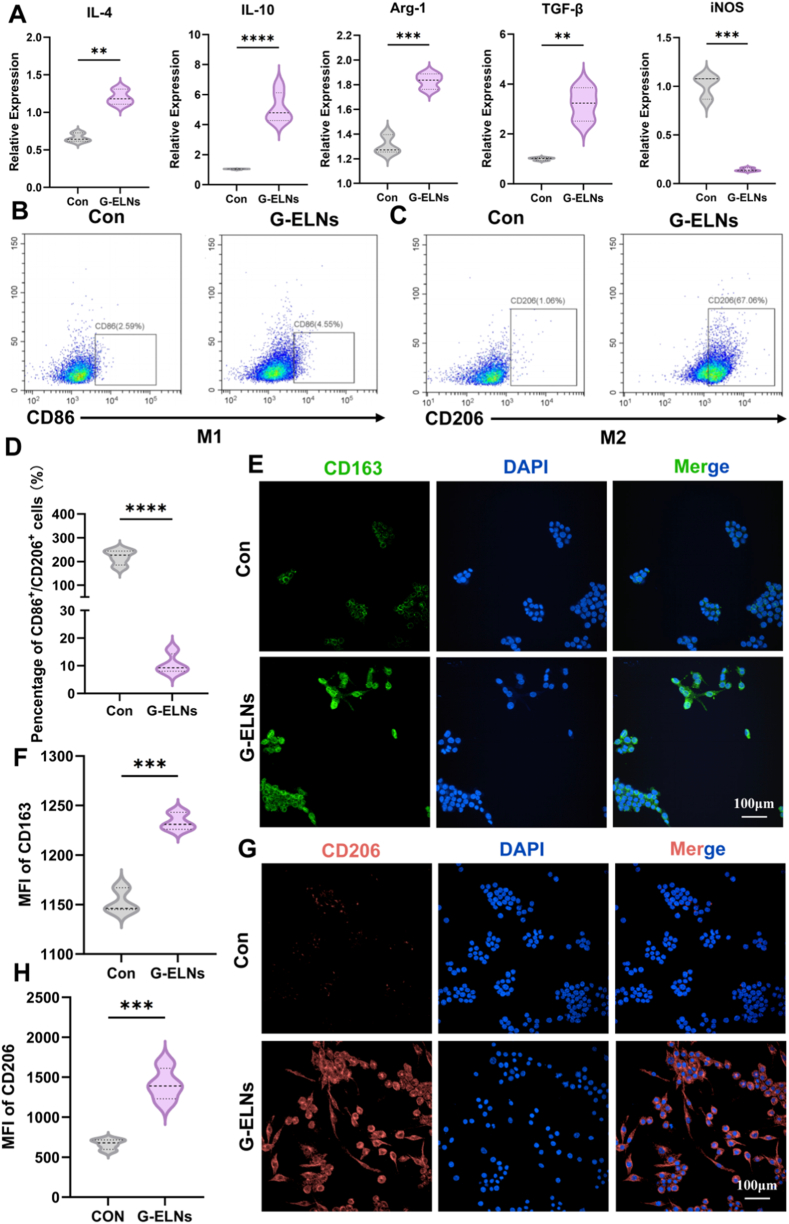
**G-ELNs promote M2 polarization of RAW264.7 macrophages in vitro.** (A) Quantitative analysis of mRNA expression levels of *Il-4, Il-10, Arg-1, Tgf-β*, and *iNOS* in RAW264.7 cells across experimental groups. (B-D) Flow cytometry analysis of M1 phenotype (CD86^+^) and M2 phenotype (CD206^+^) in RAW264.7 cells treated with G-ELNs, along with quantitative assessment. (E-F) Confocal microscopy images and quantitative analysis of M2 phenotype marker CD163^+^ in G-ELNs-treated RAW264.7 cells. (G-H) Confocal microscopy images and quantitative analysis of M2 phenotype marker CD206^+^ in G-ELNs-treated RAW264.7 cells. *ns:p*>*0.05*; *∗p < 0.1, ∗∗p < 0.01, ∗∗∗p < 0.001, ∗∗∗∗p < 0.0001.*

To evaluate the impact of G-ELNs on macrophage polarization, we conducted multi-dimensional validation through cytomorphological observation, qPCR, flow cytometry, and immunofluorescence. Fig. S4A shows that RAW264.7 cells in their normal state exhibited typical round or short spindle shapes, whereas G-ELNs treatment induced a significant morphological shift to elongated spindle shapes (Fig. S4B), consistent with M2 macrophage polarization characteristics. qPCR results revealed that, compared to the Con group, G-ELNs treated RAW264.7 cells exhibited significantly upregulated mRNA expression levels of M2 macrophage markers (*Il-4, Il-10, Arg-1, Tgf-β*) and significantly downregulated expression of the M1 marker *iNOS* (Fig. 2A). Flow cytometry analysis of the proportions of M1 (CD86^+^) and M2 (CD206^+^) macrophages in the Con and G-ELNs groups (Fig. 2B–D) demonstrated that the CD86^+^/CD206^+^ ratio was significantly downregulated in the G-ELNs group, indicating robust promotion of M2 polarization. Immunofluorescence further confirmed that RAW264.7 cells treated with 100 μg/mL G-ELNs exhibited markedly higher fluorescence intensity of M2-specific markers CD163 and CD206 compared to the Con group (Fig. 2E–H). In the in vitro model simulating diabetic hyperglycemic conditions (HG group), G-ELNs demonstrated a significant capacity to promote M2 polarization of RAW264.7 macrophages. As shown in Fig. S17A–C, qPCR analysis revealed that while M2 marker genes were substantially downregulated in the HG group versus the control (Con) group, the HG + G-ELNs group exhibited marked enhancement of these genes. Specifically, G-ELNs intervention induced systematic upregulation of key M2 markers (IL-10, Arg-1, TGF-β), with concurrent immunofluorescence staining confirming a pronounced increase in CD206+cell proportion (Fig. S17D–E). These findings collectively validate G-ELNs' ability to reverse high-glucose-induced M1 bias and reprogram macrophage phenotype toward an anti-inflammatory reparative state. The results conclusively indicate that G-ELNs effectively activate M2-polarization pathways even within pathological hyperglycemic microenvironments, providing compelling in vitro evidence for clinical translation in diabetic wound healing.

Macrophage polarization is a dynamic process modulated by microenvironmental signals, and the M1/M2 balance directly influences the inflammatory environment of wounds [36]. In diabetic wounds, the hyperglycemic environment leads to a tendency of macrophages towards M1 polarization, generating large amounts of pro-inflammatory cytokines and maintaining a chronic inflammatory state, which hinders the normal healing of wounds [37]. Therefore, regulating macrophage polarization and promoting their transformation to the M2 type is of great significance for improving the healing of diabetic wounds. The results of this study show that G-ELNs can significantly enhance the M2 polarization of RAW264.7 macrophages by up-regulating the expression of M2 markers ((*Il-4, Il-10, Arg-1, Tgf-β*)) and down-regulating the expression of M1 markers (*iNOS*). This regulatory effect holds potential clinical significance for the treatment of diabetic wounds. Specifically, IL-10, as an important anti-inflammatory cytokine, can reduce chronic inflammation by inhibiting the expression of pro-inflammatory cytokines [38]. TGF-β can promote the proliferation of fibroblasts and the synthesis of collagen, thereby accelerating the remodeling of skin tissue. In addition, TGF-β also has the function of regulating immune responses, which helps maintain immune balance during the wound healing process [39]. Arg-1 can synthesize polyamines through arginine metabolism, promoting cell proliferation and accelerating cell regeneration during the healing process of diabetic wounds [40,41]. In summary, these findings suggest that G-ELNs can reprogram the pro-inflammatory microenvironment dominated by M1 under diabetic wounds into a repair-oriented microenvironment dominated by M2, thereby coordinating the inhibition of chronic inflammation, promoting extracellular matrix reconstruction, and accelerating the entire process of tissue regeneration. To further investigate the mechanism in primary macrophages, flow cytometric analysis was performed and confirmed that the isolated BMDMs were >95% pure (F4/80^+^, Fig. S6). To validate the universality of G-ELNs in regulating primary macrophage efferocytosis and phenotypic polarization, we co-cultured PKH26-labeled apoptotic Jurkat cells with bone marrow-derived macrophages (BMDMs). Immunofluorescence results demonstrated significantly increased intracellular PKH26^+^ apoptotic cells in G-ELNs-treated BMDMs, with quantitative analysis revealing a 96% enhancement in phagocytosis rate versus controls. Moreover, G-ELNs simultaneously upregulated the efferocytosis core receptor MERTK (fluorescence intensity increased by 112% compared to Con group) and the M2 marker CD163 (52% fluorescence intensity elevation, Fig. S7). These results align with RAW264.7 model data, confirming that G-ELNs cross-modally regulate macrophage homeostasis by synergistically enhancing efferocytosis capacity and pro-repair phenotypic conversion.

### 3.3 Bioinformatics analysis of G-ELNs in regulating RAW264.7 cells

The ability of G-ELNs to promote M2 macrophage polarization suggests a potential role in improving wound repair. However, the molecular mechanisms underlying G-ELNs-mediated M2 polarization and their subsequent regulation of skin tissue repair remain largely unknown. In this study, we performed transcriptomic analysis on G-ELNs-treated RAW264.7 cells by miRNA sequencing to elucidate the mechanisms responsible for G-ELNs-induced M2 polarization and their function in skin tissue repair.

**Fig. 3:**
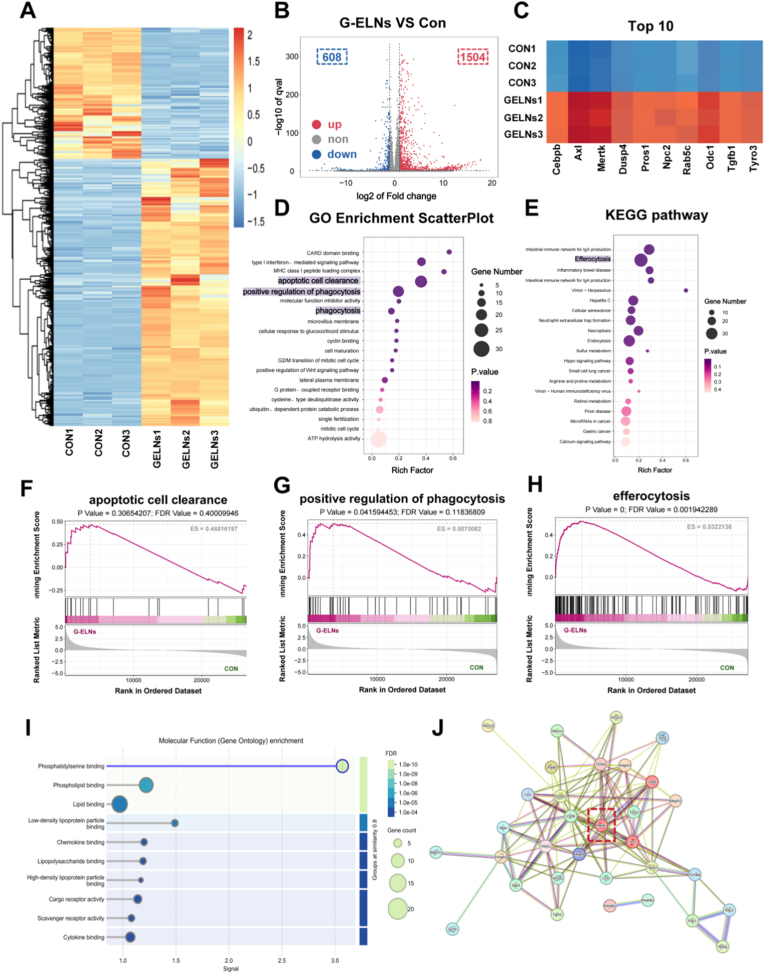
**Bioinformatics analysis of G-ELNs-treated RAW264.7 cells.** (A) Heatmap of differentially expressed genes (DEGs) between the Con and G-ELNs groups, with red and blue representing upregulated and downregulated expression, respectively. (B) Volcano plot of DEGs between the Con and G-ELNs groups. (C) Heatmap of the top 10 DEGs. (D) Gene Ontology (GO) biological process analysis. (E) KEGG pathway enrichment analysis. (F-H) Gene Set Enrichment Analysis (GSEA) of efferocytosis-related GO terms and KEGG pathways. (I) Molecular function enrichment map for efferocytosis-related genes, highlighting predominant enrichment in phosphatidylserine (PS) binding. (J) STRING protein-protein interaction (PPI) network for efferocytosis-related molecules, with TAM family receptors (Axl, Tyro3, MerTK) identified as classical PS receptors.

The heatmap of differentially expressed genes (DEGs) (Fig. 3A) and volcano plot (Fig. 3B) clearly illustrated transcriptional differences between groups, from which we identified a total of 1504 up-regulated and 608 down-regulated genes. These extensive transcriptomic alterations demonstrate that G-ELNs induce transcriptome reprogramming in macrophages, likely by activating specific signaling networks. The heatmap of the top 10 DEGs showed that the expression of inflammation regulation or phagocytosis related genes was markedly upregulated, including *Mertk*, *Axl* and *Pros1* (Fig. 3C), which might be the key regulatory nodes. Gene Ontology (GO) biological process analysis showed that DEGs were mostly enriched in “phagocytosis” (Fig. 3D), “apoptotic cell clearance” and “regulation of phagocytosis”, which were critical processes for macrophages to remove necrotic cells and maintain microenvironmental homeostasis during the process of wound healing. KEGG pathway enrichment analysis further localized the “efferocytosis” pathway (Fig. 3E), whose critical roles in coordinating inflammation resolution and tissue regeneration provided important clues for G-ELNs pro repair mechanism. GSEA analysis revealed significant enrichment of efferocytosis gene sets enrichment in the G-ELNs group (Fig. 3F–H), suggesting that G-ELNs regulate efferocytosis, a highly conserved process for the maintenance of tissue homeostasis by mediating apoptotic cell clearance. Efferocytosis promotes repair by restraining chronic inflammation and releasing reparative cytokines (IL-10, TGF-β) linking inflammation resolution and regeneration [42,43]. Through functional enrichment maps and STRING protein-protein interaction (PPI) network, we identified molecular targets of G-ELNs in efferocytosis. GO molecular function analysis showed that efferocytosis-related genes were mainly enriched in “phosphatidylserine (PS) binding” (Fig. 3I) and PS was one of “eat-me” signals exposed on the surface of apoptotic cells. When binding to the receptors on macrophages, PS triggered efferocytosis (Fig. 3I). Furthermore, the STRING PPI network displayed that TAM family receptors (MERTK, Axl, Tyro3) and their ligand Gas6 were the main core interactors (Fig. 3J), and MERTK had the highest connectivity. Previous studies have established that MERTK, a key member of the TAM family, serves not only as a critical macrophage receptor for phosphatidylserine recognition and efferocytosis initiation but also as a specific marker for the M2c subtype [44,45]. This evidence suggests that MERTK is likely a pivotal target through which G-ELNs regulate the efferocytosis process in M2c macrophages.

### 3.4 G-ELNs up-regulate MERTK expression and enhance efferocytosis in M2c macrophages In vitro

**Fig. 4:**
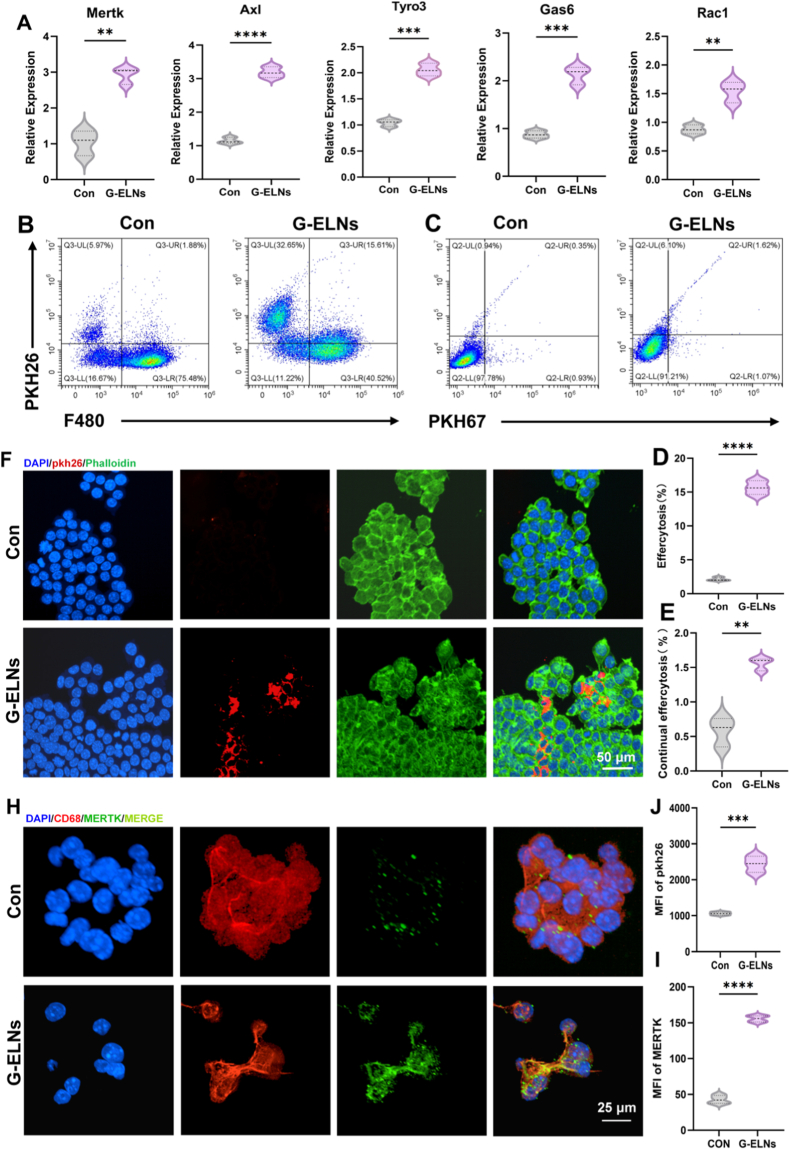
**G-ELNs upregulate MERTK expression and enhance efferocytosis in M2c macrophages in vitro.** (A) Quantitative analysis of mRNA expression levels of Mertk, Axl, Tyro3, Gas6, and Rac1 in RAW264.7 cells across experimental groups. Data parameters as Fig. 2A. (B, D) Flow cytometry analysis and quantitative assessment of efferocytosis in G-ELNs-treated RAW264.7 cells. Data from 3 independent experiments (n = 3 experiments), 3 biological replicates per group. (C, E) Flow cytometry analysis and quantitative assessment of sustained efferocytosis (sequential apoptotic cell clearance) in G-ELNs-treated RAW264.7 cells. Data from 3 independent experiments (n = 3 experiments), 3 biological replicates per group. (F, J) Confocal microscopy images and quantitative analysis of efferocytosis in G-ELNs-treated RAW264.7 cells. Data parameters as Fig. 2E–H. (H, I) Confocal microscopy images and quantitative analysis of MERTK protein expression in G-ELNs-treated RAW264.7 cells. Data from 3 independent experiments (n = 3 experiments), 3 biological replicates per group. *ns:p*>*0.05*; *∗p < 0.1, ∗∗p < 0.01, ∗∗∗p < 0.001, ∗∗∗∗p < 0.0001.*

Building on prior transcriptomic findings, we further investigated the mechanism by which G-ELNs modulate efferocytic function in M2c-subtype macrophages. During apoptotic cell model construction, Jurkat cells subjected to 1.5-h UVC irradiation achieved an apoptosis rate exceeding 80% (Fig. S5), providing standardized target cells for subsequent efferocytosis assays. qPCR analysis revealed that G-ELNs-treated RAW264.7 cells exhibited significantly upregulated mRNA levels of efferocytosis-related genes (*Mertk, Axl, Tyro3, Gas6, Rac1*) compared to the Con group (Fig. 4A). Interestingly, *Mertk* was significantly up-regulated as it is a receptor for efferocytosis and a typical marker of M2c macrophages. This result is consistent with the transcriptome results we analyzed earlier. In addition, our qPCR and immunofluorescence experiments showed that G-ELNs markedly upregulated M2c secretory factors (*Il-10*, *Tgf-β*) and CD163 fluorescence intensity (Fig. 2A–E, F). These results further demonstrated that G-ELNs could robustly induce M2c polarization and enhance efferocytic capacity in macrophages. Subsequently, we also used flow cytometry to quantify the efferocytosis levels. The results showed that G-ELNs treated RAW264.7 cells significantly enhanced phagocytosis of PKH26-labeled apoptotic Jurkat cells (from 1.88% to 15.61%, Fig. 4B–D). In the sustained efferocytosis assay, G-ELNs treated macrophages could significantly increase the level of sustained efferocytosis. Compared with the Con group, the continuous apoptotic cell clearance of G-ELNs group was increased by 4 times (Fig. 4C–E). In addition, the co-culture of PKH26^+^ apoptotic Jurkat cells and RAW264.7 macrophages was used for immunofluorescence observation. The results showed that the fluorescence intensity of PKH26 debris in the intracellular was increased by 117.8% in the G-ELNs group (Fig. 4F–J), and directly observed the phenomenon of enhanced efferocytosis. In order to verify the effect of MERTK, which was identified as a key protein in efferocytosis network, we also conducted immunofluorescence staining.

The results showed that the fluorescence intensity of MERTK protein was increased by 212.5% in G-ELNs group (*P < 0.001*, Fig. 4H–I). As shown in the above, the efferocytosis could efficiently clear apoptotic cell debris. The impairment of efferocytosis could cause the accumulation of necrotic cells and induce chronic inflammation. The above functional experiments clearly demonstrated that G-ELNs could enhance efferocytic capacity and sustained efferocytic activity in M2c macrophages via upregulating MERTK expression. In diabetic wounds, the apoptotic cell burden was high and the sustained efferocytic capability was necessary to break the “inflammation-apoptosis” cycle [42,46,47]. These results were also consistent with the results of bioinformatics analysis, which provided the molecular basis for the subsequent in vivo experiments.

To determine whether G-ELNs enhance efferocytosis through specific activation of MERTK, we screened for the optimal concentration of the MERTK inhibitor using CCK-8 and Western Blot assays. The CCK-8 results showed that UNC2025 concentrations below 100 nM had no significant cytotoxicity on macrophage viability (Fig. S8). Western Blot analysis revealed that 10 nM UNC2025 effectively inhibited MERTK protein expression. Therefore, 10 nM UNC2025 was selected for subsequent functional blockade experiments (Fig. S9).

In the MERTK functional blockade assay, compared with the control group, the G-ELNs group exhibited significantly upregulated expression of MERTK and its downstream effector Arg 1. However, this upregulation induced by G-ELNs was markedly attenuated in the presence of the UNC2025 inhibitor. These results demonstrate that inhibiting MERTK activity effectively blocks the downstream effects of G-ELNs, confirming that G-ELNs specifically activate the Mertk receptor to upregulate Arg 1 expression and thereby enhance efferocytosis (Fig. S10).

### 3.5 Preparation, characterization, and biocompatibility evaluation of SM hydrogel

**Fig. 5:**
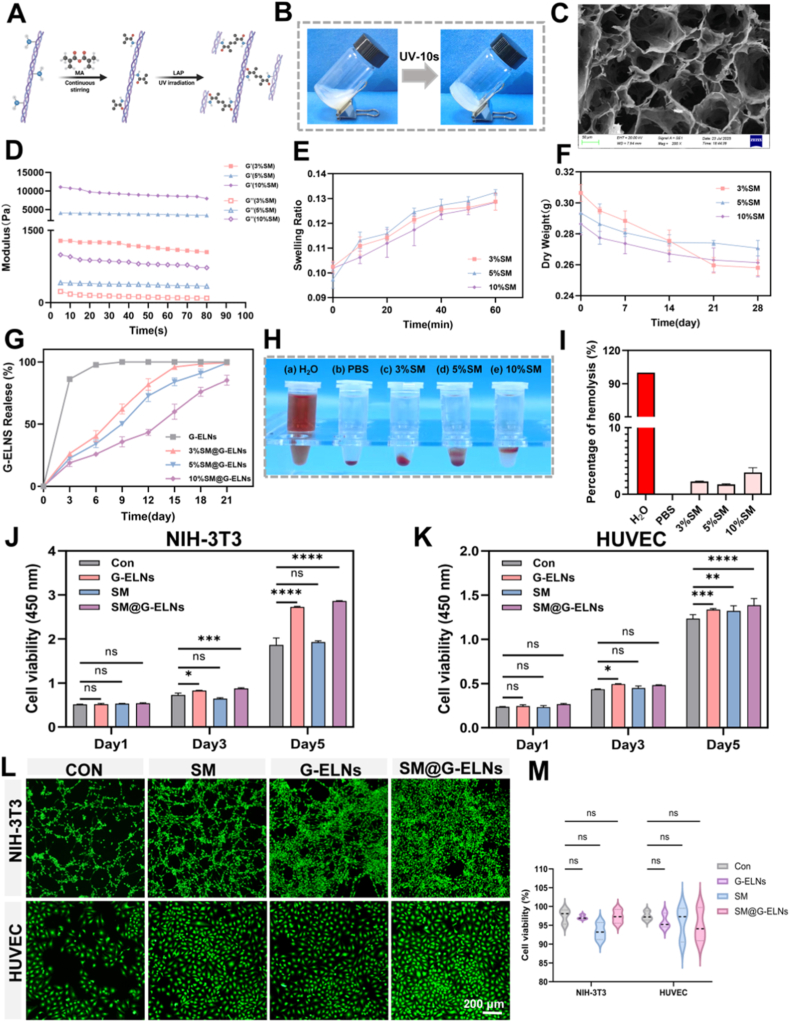
**Preparation, characterization, and cytocompatibility evaluation of SM hydrogel.** (A) Schematic illustration of SM hydrogel synthesis. (B) Gelation process images. (C) SEM image. (D) Rheological profiles (storage modulus G′ and loss modulus G″) of 3%, 5%, and 10% SM hydrogels. (E) Swelling kinetics of SM hydrogels. (F) Degradation curves of SM hydrogels in PBS. (G) Cumulative release profile of G-ELNs from SM hydrogel. (H, I) Hemolysis assay images and hemolysis rate quantification for SM hydrogels at varying concentrations. (J, K) Proliferation of NIH-3T3 fibroblasts and HUVECs co-cultured with extracts from different SM hydrogel groups for 1, 3, and 5 days, measured via CCK-8 assay. (L, M) Live/dead staining images and quantification of NIH-3T3 and HUVEC cells after 3 days of incubation with SM hydrogel extracts.The above data from 3 independent experiments (n = 3 experiments), 3 biological replicates per group, 3 technical replicates per sample. *ns:p*>*0.05*; *∗p < 0.1, ∗∗p < 0.01, ∗∗∗p < 0.001, ∗∗∗∗p < 0.0001.*

Building on our previous research, we prepared the SM hydrogel [16]. The fabrication principle is illustrated as follows: porcine small intestinal submucosa (SIS) was treated via acid digestion to fully expose surface amino groups, which underwent amidation with carboxyl groups in methacrylic anhydride (MA) to form a stable covalent crosslinked network. In this system, SIS not only serves as a three-dimensional structural scaffold providing mechanical support but also gains photo-responsive properties through introduced methacryloyl groups, enabling secondary crosslinking via UV light to modulate pore architecture. Macroscopically, the SM hydrogel showed semitransparent and homogeneous morphology with no delamination. When contacting with 405 nm UV light, the SM liquid rapidly converted into gel state in 10 s (Fig. 5B). The successful grafting of methacryloyl groups onto SIS backbone and the thorough removal of unreacted small molecules is confirmed by 1H NMR spectra (Fig. S11). SEM analysis further demonstrated that the SM hydrogel possessed interconnected porous structure with pore size of 50 ∼ 120 μm (Fig. 5C), which was beneficial for nutrient exchange and G-ELNs encapsulation/delivery.

Rheological experiments indicated that the storage moduli (G′) of SM hydrogels at 3%, 5%, and 10% were much larger than loss moduli (G″) (Fig. 5D), implying that the SM hydrogels had strong mechanical properties to resist wound pressure. Further Large Amplitude Oscillatory Shear (LAOS) analysis revealed that the 5% SM hydrogel possessed a comparable but slightly softer viscoelastic profile (G' ∼3-4 kPa) than native porcine skin (G' ∼10 kPa) (Fig. S12), which is highly desirable for conformal adaptation to irregular diabetic wounds without causing secondary mechanical trauma. The swelling experiment indicated that the SM hydrogels reached equilibrium swelling in PBS in 60 min, and the swelling ratio of 5% group reached the highest (13.1%) (Fig. 5E), which was suitable for absorbing exudate and creating moist microenvironment.

The result of G-ELNs degradation in PBS indicated that the SM hydrogels groups of 3% and 5% exhibited gradual degradation in PBS, and the group of 3% SM degraded by 50% in 14 days (Fig. 5F). The morphological evolution during degradation was further confirmed by SEM observation over a 21-day period (Fig. S13). While the groups of 5% and 10% had degraded completely in 28 days, which coincided with diabetic wound healing timeline (Fig. 5F). The drug release experiment indicated that G-ELNs loaded SM hydrogels could achieve cumulative drug release rate of ∼75% in 14 days, and the release process could be divided into two stages, i.e., a rapid release stage (∼30% in 24 h) and a long-term release stage (Fig. 5G). TEM results demonstrate that both before and after UV irradiation, G-ELNs exhibit typical vesicular structures with consistent vesicle morphology and size distribution, maintaining structural integrity (Fig. S16). This observation corroborates the stability of encapsulation efficiency and kinetic profiles in the release curves, confirming that the photopolymerization process does not compromise the bioactivity of G-ELNs, thereby ensuring their reliability as drug carriers. Moreover, the long-term release tracking indicated that the free G-ELNs in PBS were released 89.1% in 3 days, while G-ELNs loaded in 5% SM hydrogel was almost released (98.4%) in 28 days, compared with 83.7% in 10% group (Fig. 5H). This appropriate release profile could avoid the situation that drug was depleted prematurely (3% group) or retained for too long (10% group) (Fig. 3F), and therefore, SM hydrogel could be used as sustained intelligent release delivery system.

Biocompatibility tests revealed that hemolysis levels were <5% for all SM concentrations (<5% RBC hemolysis, Fig. 5H–I) and met the requirements for biomaterials. Therefore, we used the 5% SM hydrogel for subsequent cell and in vivo studies. While the release kinetics were strictly quantified via the aforementioned direct release tracking, the CCK-8 assay was specifically utilized to evaluate the bioactivity and safety of the system. NIH-3T3 fibroblasts and HUVECs cultured with G-ELNs, SM hydrogel extracts, or SM@G-ELNs extract for 3 ∼ 5 days showed significantly higher cell viabilities in the SM@G-ELNs and G-ELNs groups compared with those in the Con group (Fig. 5J–K). This result not only confirmed the excellent time-dependent biocompatibility of the SM hydrogel, but also verified that the continuously released G-ELNs successfully retained their pro-proliferative bioactivity over time. Live/dead staining further demonstrated that cells in all treatment groups exhibited good proliferation and survival capacities (Fig. 5L). The physicochemical properties of SM hydrogel meet the needs of diabetic wound repair as follows: its interconnected porous structure facilitates G-ELNs distribution/release and cell migration; mechanical stability prevents structural collapse while shielding against infection; and controlled swelling/degradation kinetics match healing phases, avoiding premature G-ELNs depletion or delayed tissue remodeling [16,[48], [49], [50]].

### 3.6 SM hydrogel loaded with G-ELNs significantly accelerates diabetic wound healing in rats

The aforementioned findings demonstrated the potential of G-ELNs to promote M2c macrophage polarization and regulate efferocytosis, coupled with the excellent biocompatibility of SM hydrogel. We subsequently established a full-thickness diabetic skin defect model in SD rats to evaluate the therapeutic efficacy of the SM@G-ELNs system in vivo. A type 1 diabetic model was successfully induced via intraperitoneal streptozotocin (STZ) injection (blood glucose >16.7 mmol/L sustained for 4 weeks), mimicking impaired diabetic wound healing (characteristic symptoms: polydipsia/polyphagia, polyuria, and lethargy). A 10-mm-diameter full-thickness wound was created on the dorsum, and rats were randomized into five groups: Sham-operated group (Sham, diabetic but did not receive any surgery or treatment), control group (Con, diabetic untreated), blank hydrogel group (SM), free G-ELNs group (G-ELNs), and SM@G-ELNs hydrogel group.

**Fig. 6:**
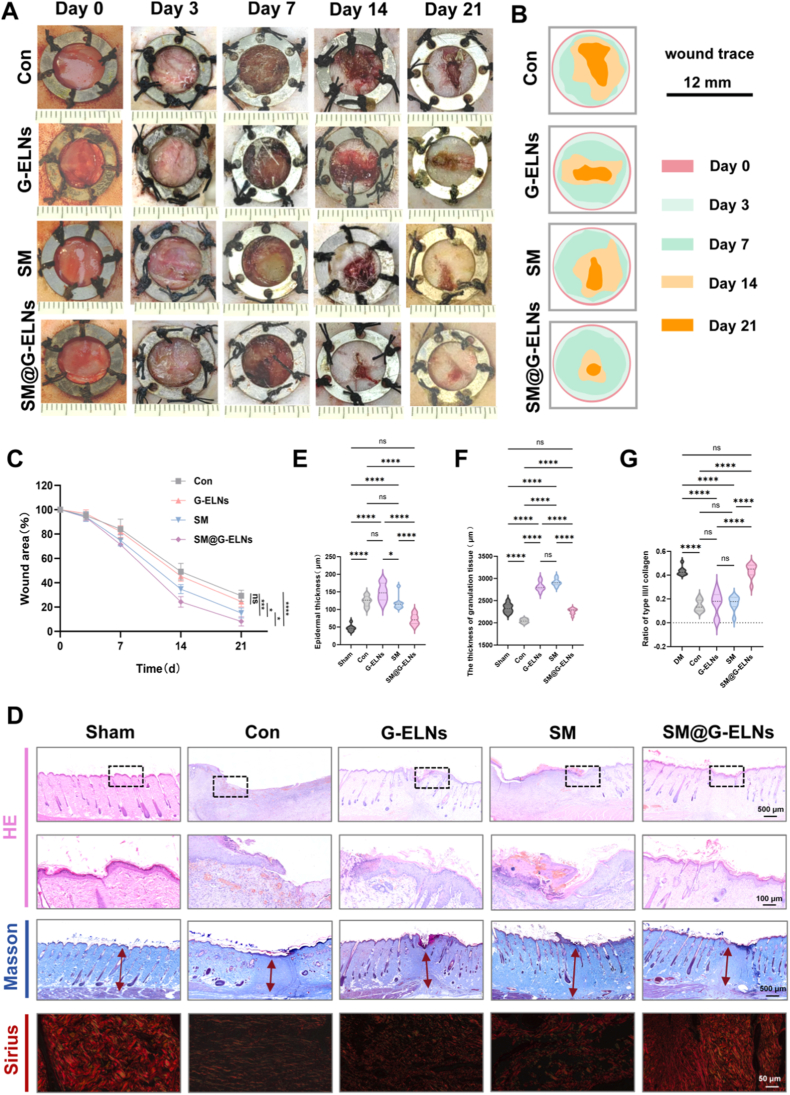
**In vivo evaluation of diabetic wound healing performance using SM hydrogel loaded with G-ELNs.** (A) Macroscopic images of wound closure progression. (B) Wound healing trajectory mapping. (C) Quantitative analysis of wound area reduction over time. (D) Representative histopathological images of H&E, Masson's trichrome, and Sirius red staining on day 21 post-treatment. (E-G) Statistical quantification of epidermal thickness, dermal thickness, and type ⅠII/I collagen ratio. (n = 8), *ns:p*>*0.05*; *∗p < 0.1, ∗∗p < 0.01, ∗∗∗p < 0.001, ∗∗∗∗p < 0.0001.*

Macroscopic observations revealed significantly faster wound closure rates in the SM@G-ELNs group at all time points (3, 7, 14, 21 days) compared to other groups (Fig. 6A). Quantitative analysis of wound area percentages showed that by day 21, healing rates in the Control and G-ELNs groups were only 63.8% and 70.6%, respectively, whereas the SM and SM@G-ELNs groups achieved 83.2% and 97.4% closure (Fig. 6B–C), with SM@G-ELNs nearing complete regeneration.

H&E staining indicated inflammatory responses across all groups at days 3-7. By day 14, the SM@G-ELNs group showed accelerated regeneration: fast thickening of the epidermal stratum corneum, which formed a continuous layer, orderly arrangement of fibroblasts in the dermis, and regeneration of skin appendages (Fig. S14). By day 21, SM@G-ELNs showed mature histological phenotypes such as complete stratified epidermis, a high number of neogenic hair follicles/sweat glands, and little inflammatory infiltration compared with Con. At 21 days, compared with the con group, the SM@G-ELNs group showed mature histological phenotypes such as complete epidermis, more new hair follicles/sweat glands, and less inflammatory infiltration. The thickness of epidermis (SM@G-ELNs, 71.55 μm) and dermis (SM@G-ELNs, 2257.94 μm) was closest to that of Sham group (50.37 μm, 2331.71 μm). Polarized light microscopy of Sirius red-stained collagen revealed that collagen in SM@G-ELNs was arranged in a braid/basket fiber pattern (Fig. 6D) similar to normal skin (Sham group), whereas collagen in Con, G-ELNs, and SM was arranged in parallel bundles, characteristic of pathological scars. Compared with Con, SM@G-ELNs significantly increased the ratio of type III/I collagen to the level of Sham (Fig. 6G, Fig. S18). Collagen deposition in diabetic wounds is often inadequate and the collagen maturity is low. An increased type III/I collagen ratio improves mechanical strength and decreases the risk of scarring by enhancing the proportion of physiological subtypes of collagen. Our results show that SM@G-ELNs do not only promote collagen synthesis, but also maintain an appropriate proportion of physiological subtypes of collagen, promoting functional and mature tissue regeneration [51].

### 3.7 SM hydrogel loaded with G-ELNs enhances efferocytosis by upregulating MERTK expression in diabetic wounds

**Fig. 7:**
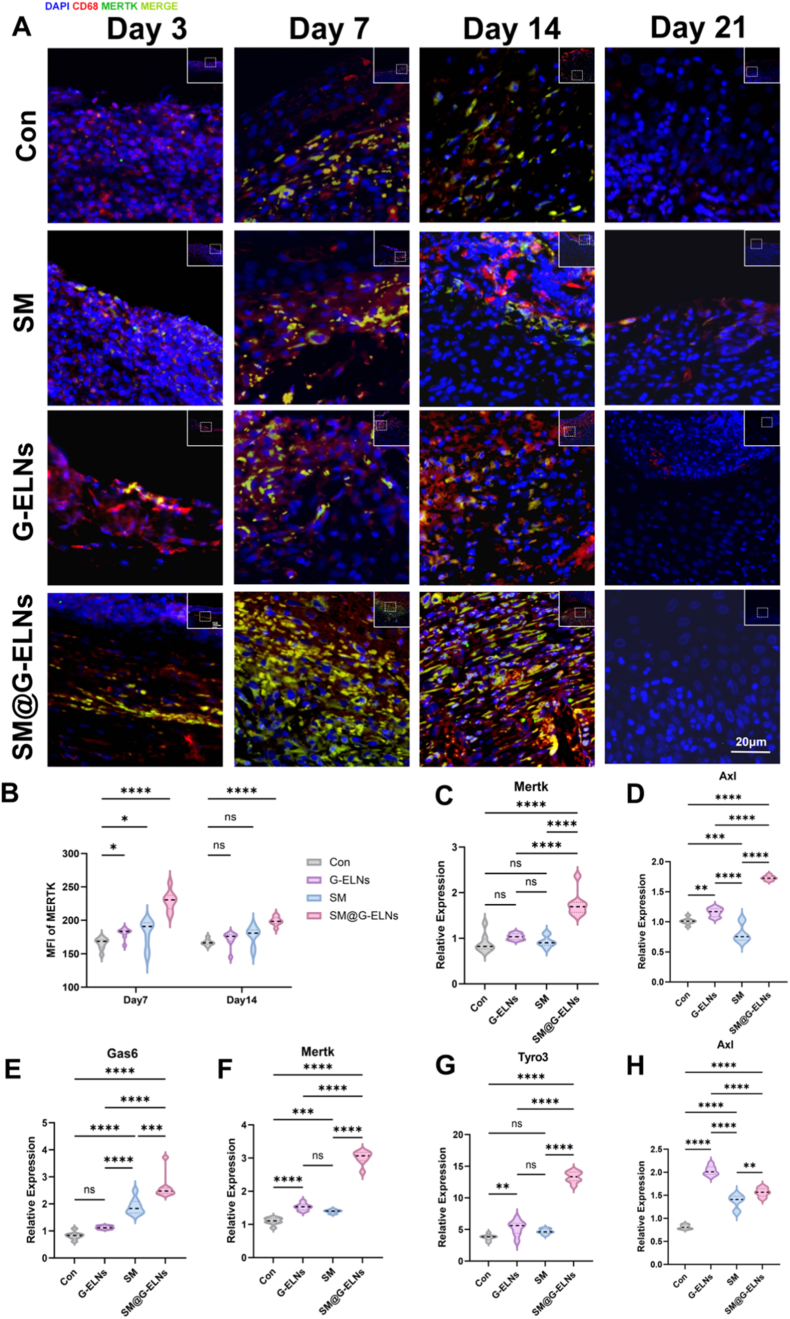
**SM hydrogel loaded with G-ELNs promotes diabetic wound healing in rats by upregulating MERTK protein expression in M2c-subtype macrophages to enhance efferocytosis.** (A) Immunofluorescence staining of CD68 and MERTK in wound tissues at days 3, 7, 14, and 21 post-treatment. (B) Quantitative analysis and statistics of CD68^+^/MERTK^+^double-positive cells at days 7 and 14. (C-E) mRNA expression levels of Mertk, Axl, and Gas6 in skin tissues across groups at day 7. (F-H) mRNA expression levels of Mertk, Tyro3, and Axl in skin tissues across groups at day 14. (n = 8), *ns:p*>*0.05*; *∗p < 0.1, ∗∗p < 0.01, ∗∗∗p < 0.001, ∗∗∗∗p < 0.0001.*

Combining histological results, we found that SM@G-ELNs not only promoted reepithelialization by accelerating the process but also guided the formation of collagen fiber as the structural basis of skin barrier recovery. To further prove the regulatory effect of SM@G-ELNs system on upregulating efferocytosis in vivo, we initially analyzed the expression of MERTK in macrophages of wound tissues by immunofluorescence. Results showed that the number of double positive cells of CD68^+^/MERTK^+^ in SM@G-ELNs group at different postoperative time points was significantly higher than that in Con group. The maximum value was reached at day 7 (Fig. 7A). The number of double positive cells in SM@G-ELNs group at day 7 was 69.4% higher than that in Con group (P < 0.0001, Fig. 7B). Upregulation of MERTK, an important receptor of efferocytosis, in macrophages provides direct evidence at the molecular level for the enhanced efferocytic effect of SM@G-ELNs. Interestingly, the expression of MERTK reached the peak at early proliferative phase (day 7), which coincided with the need of apoptotic cell clearance, further confirming the time-dependent regulatory effect in wound repair.

At transcriptional level, the system not only upregulated Mertk but also synergistically activated its family members Axl, Tyro3, and its ligand Gas6 (Fig. 7C–H). The broad modulation of TAM signaling in system is consistent with that in vitro, which is also mechanistically reasonable at two experimental models. Importantly, the high expression of MERTK maintained at later remodeling phase (day 14) suggests that SM@G-ELNs induces prolonged biological effects at later stage, which maintains efficient efferocytosis even at later stage of tissue restructuring and prevents the occurrence of secondary inflammation induced by apoptotic cell accumulation, thereby creating pro-regenerative microenvironment for high quality healing.

**Fig. 8:**
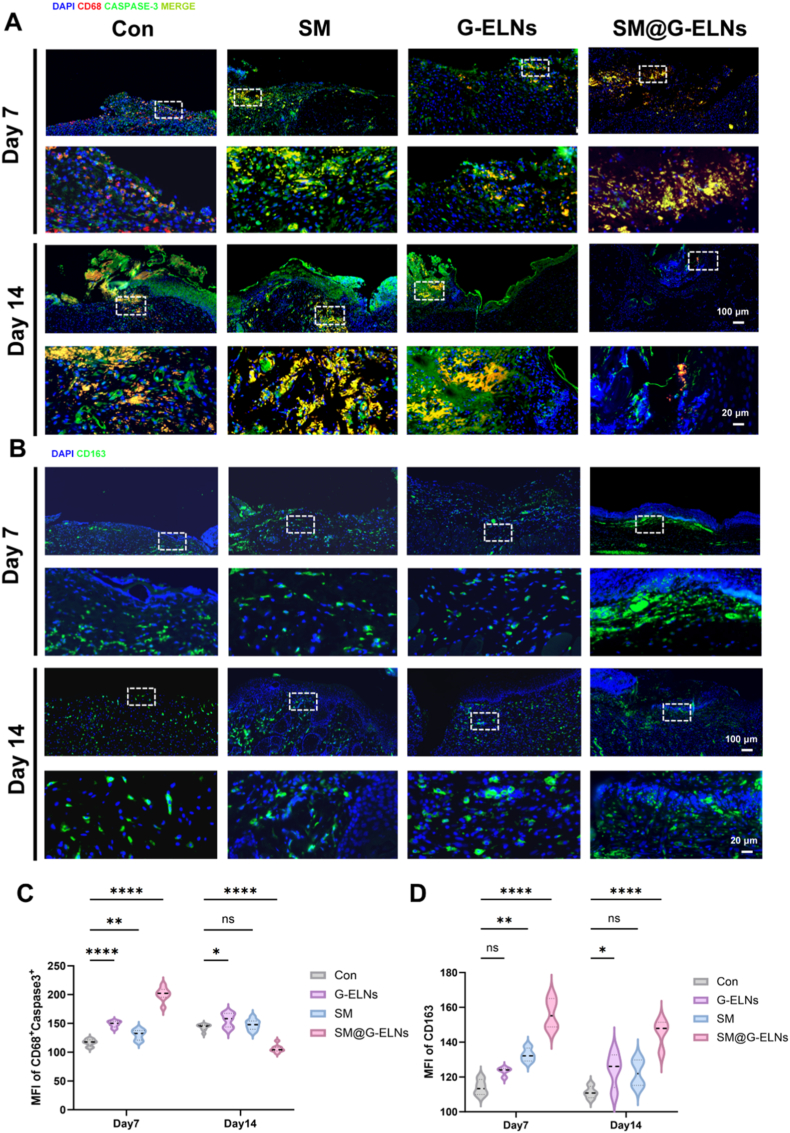
**SM hydrogel loaded with G-ELNs promotes diabetic wound healing in rats by enhancing efferocytosis.** (A) Immunofluorescence staining of CD68 and Caspase-3 in wound tissues at days 7 and 14 post-treatment. (B) Immunofluorescence staining of CD163 in wound tissues at days 7 and 14. (C, D) Quantitative analysis and statistical results of CD68^+^/Caspase-3^+^ double-positive cells and CD163^+^ macrophages. Data parameters as Fig. 7B *ns:p*>*0.05*; *∗p < 0.1, ∗∗p < 0.01, ∗∗∗p < 0.001, ∗∗∗∗p < 0.0001.*

The expression of efferocytosis-related genes was detected by qPCR. SM@G-ELNs upregulated the expression of Mertk mRNA at day 7 by 0.75-fold, Axl by 0.77-fold, and Gas6 by 1.23-fold compared with Con (Fig. 7C–E). Notably, at day 14, Mertk expression in the SM@G-ELNs group remained 2.15-fold higher than Con (P < 0.001), with concurrent upregulation of Tyro3 and Axl (Fig. 8F–H). These results mirror in vitro data, demonstrating that G-ELNs activate efferocytosis via the same molecular mechanism in vivo: upregulating TAM family receptors and ligands to amplify signaling. Persistent MERTK activation at day 14 underscores the hydrogel's ability to sustain efferocytosis during tissue remodeling, ensuring thorough apoptotic debris removal and optimizing regenerative outcomes [41,[52], [53], [54]]. To further validate the immunohistochemical findings, we quantitatively assessed MERTK protein expression in wound tissues from each treatment group at day 14 post-treatment by Western blot analysis. As shown in Fig. S19, the SM@G-ELNs group exhibited the highest MERTK protein expression among all groups, followed by the SM and G-ELNs groups, while the control group showed the lowest expression level. These results are consistent with the increased number of CD68^+^/MERTK^+^ double-positive cells observed in the SM@G-ELNs group at day 14, further confirming that SM@G-ELNs treatment enhances MERTK expression at the protein level.

### 3.8 SM hydrogel loaded with G-ELNs promotes wound healing by enhancing local efferocytosis

TUNEL staining (Fig. S15) revealed significantly lower apoptotic cell fluorescence intensity in the SM@G-ELNs group at postoperative day 7 compared to Con, suggesting that the material mitigates tissue damage by suppressing excessive apoptosis, thereby creating favorable conditions for re-epithelialization. To further elucidate the underlying mechanism, we dynamically analyzed macrophage phenotype switching and efferocytosis efficiency at critical time points (days 7 and 14) via immunofluorescence (Fig. 8).

Immunofluorescence and quantification. In wound tissues in the proliferative phase (days 7-14 after injury) (Fig. 8B–D), the proportion of CD163+ M2c macrophages infiltrating the wound tissue in the SM@G-ELNs hydrogel group was significantly higher than that in Con on days 7 and 14 after injury. The proportion of CD163+ M2c macrophages increased by 36.5% on day 14 after injury compared with that in the Con group (*P < 0.001*), which is consistent with the stage-specific regulation of macrophage phenotypes during the process of wound healing.

Previous studies have reported that after the resolution of inflammation, the proliferative phase is another critical time window during repair, in which M2c macrophages dominate, these macrophages promote the repair process by accelerating tissue regeneration through the regulation of pro-angiogenic factor secretion and the regulation of collagen deposition [35]. The SM@G-ELNs hydrogel improved the efficiency of repair in the proliferative phase by enhancing the effect of M2c polarization, further accelerating the healing process.

Compared with Con, immunofluorescence analysis at day 7 after injury showed that the CD68^+^/Caspase-3^+^ double positive cell population in the SM@G-ELNs group was significantly increased (Fig. 8A). Quantitative analysis showed that the proportion of CD68+/Caspase-3^+^ double positive cells increased by 65.7% in the SM@G-ELNs group compared with that in the Con group (*P < 0.001*, Fig. 8C), indicating strong local efferocytosis (Fig. 8). With the further progress of the wound healing process to day 14, the population of CD68/Caspase-3^+^ cells in the SM@G-ELNs group decreased significantly, and the Con, SM, and G-ELNs groups had an increased population of CD68^+^/Caspase-3^+^ double positive cells compared with those at day 7. In summary, we found that there was also a clear and distinct stage-specific regulatory pattern in the SM@G-ELNs treatment group: during the early stage of proliferation, the system focuses on local efferocytosis to clear apoptotic debris and eliminate obstacles for regeneration; during the later stage, the activity of efferocytosis decreases, but the population of M2 macrophages continues to increase.

To further investigate the active components of G-ELNs that promote efferocytosis, metabolomic analysis was performed on G-ELNs, with L-malic acid being the most abundant. Based on this analysis, L-malic acid was identified as its primary bioactive component, and recent studies have demonstrated that L-malic acid exhibits potent immunomodulatory functions. Specifically, L-malic acid activates the proton-sensing receptor GPR4 on the macrophage surface, triggering a signaling cascade that: (1) suppresses NF-κB-mediated release of pro-inflammatory cytokines (e.g., TNF-α, IL-6), and (2) enhances IL-10 expression through STAT3 phosphorylation, thereby polarizing macrophages toward the anti-inflammatory M2 phenotype. This M2 polarization is critical for efferocytosis, as M2 macrophages overexpress efferocytosis receptors (MERTK, AXL), which specifically recognize phosphatidylserine (PS) on apoptotic cells to initiate clearance. The execution of efferocytosis itself initiates a positive feedback loop where phagocytosed apoptotic cells stimulate macrophages to secrete IL-4/IL-13 and TGF-β, which in turn drive M2 polarization and cellular proliferation, with this self-reinforcing cycle substantially expanding efferocytic capacity and promoting macrophage population expansion, ultimately accelerating diabetic wound healing by alleviating inflammation and promoting angiogenesis, indicating that the primary drivers of macrophage proliferation and enhanced efferocytosis are L-malic acid-mediated metabolic reprogramming, rather than miRNA delivery [15,[55], [56], [57]] (Table S2.).

This finding indicates that SM@G-ELNs can dynamically adapt to the demands of different repair phases and do not monotonously activate a single pathway. Its mechanism operates through a metabolite-centric program executing "clear-first, rebuild-later" strategy: L-malic acid—the dominant bioactive component of G-ELNs—initiates early inflammation resolution by activating macrophage GPR4-STAT3 signaling to suppress pro-inflammatory cytokines (e.g., TNF-α, IL-6) and enhance IL-10-driven M2 polarization, thereby clearing inflammation to provide a regenerative microenvironment. This metabolic reprogramming prioritizes efferocytosis clearance via M2-overexpressed receptors (MERTK/AXL) that remove apoptotic debris, while executed efferocytosis triggers a self-reinforcing loop of IL-4/IL-13-TGF-β secretion to expand M2 populations. Subsequently, the program redirects cellular energy toward long-term rebuilding tasks such as extracellular matrix remodeling and angiogenesis, optimizing bioenergetic efficiency and stabilizing the repair phenotype for high-quality tissue regeneration [[58], [59], [60]].

## 4 Conclusions

This work comprehensively unveiled the key role and molecular mechanism by which the SM@G-ELNs synergistic therapeutic system promotes diabetic wound repair via in vitro cellular experiments, animal model validation, and further bioinformatics analysis. The novelty of our work lies in the metabolite-driven "dual regulatory" effect of G-ELNs: Metabolomic analysis identified L-malic acid as the dominant component, which activates macrophage GPR4-STAT3 signaling to promote the MERTK pathway and drive M2c-subtype polarization, upregulating anti-inflammatory/pro-repair factors (e.g., IL-10, TGF-β). This reprogramming converts the wound microenvironment from chronic inflammation to pro-regeneration. Crucially, L-malic acid-initiated efferocytosis triggers a self-reinforcing loop where phagocytosed apoptotic cells stimulate IL-4/IL-13-TGF-β secretion, further amplifying M2c polarization and efferocytic capacity, thereby interrupting the "inflammation-apoptosis-secondary necrosis" cycle through metabolic reprogramming rather than miRNA delivery. To solve rapid exosome clearance, SM hydrogel enables sustained G-ELNs release via its porous structure and controllable degradation.

In summary, this work revealed a novel regulatory mechanism of G-ELNs promoting macrophage M2c efferocytosis through MERTK, and established SM@G-ELNs system as a safe, effective, and clinically promising synergistic system for treatment of diabetic wounds, with important theoretical and application significance.

## CRediT authorship contribution statement

**Yue-Qi Zhang:** Writing – original draft, Conceptualization, Investigation. **Rong Nie:** Writing – review & editing, Investigation. **Zi-Yuan Feng:** Visualization, Investigation. **Ming-Hui Fan:** Methodology. **Zhi-Xue Shen:** Investigation. **Xiu-Zhen Zhang:** Supervision. **Ji-Ye Zhang:** Investigation. **Yan-Lin Jiang:** Methodology. **Qing-Yi Zhang:** Project administration. **Kai Huang:** Visualization. **Li-Ping Mou:** Investigation. **Yan-Ming Chen:** Investigation. **Hui-Qi Xie:** Writing – review & editing, Funding acquisition, Conceptualization.

## Ethics approval and consent to participate

Animal experiments were conducted in accordance with the guidelines issued by the National Institutes of Health and were approved by the Ethics Committee of West China Hospital, Sichuan University (No. 20221201005).

## Declaration of competing interest

The authors declare that they have no known competing financial interests or personal relationships that could have appeared to influence the work reported in this paper.
