# Supplementary material for: Activating the cellular scavenger: A bioactive hydrogel promotes diabetic wounds via plant exosome-like nanovesicles enhanced macrophage efferocytosis
Source: Bioact Mater. 2026 Mar 24;62:669–85. doi: 10.1016/j.bioactmat.2026.03.039 (PMC13049916; doi:10.1016/j.bioactmat.2026.03.039)
Supplement: Multimedia component 1 [file mmc1.docx]

**Supplementary Material**

**1.Isolation, Purification, and Characterization of Grape Exosome-like Nanovesicles (G-ELNs)**

Fresh Kyoho grape pulp was homogenized in ice-cold PBS (1:3 w/v) at 5000 rpm for 5 min. The homogenate was filtered through sterile gauze and sequentially centrifuged (300×g, 4°C, 10 min; 2000×g, 20 min; 10000×g, 45 min) to remove debris. The supernatant was ultracentrifuged (100,000×g, 4°C, 70 min; Beckman Optima XE-90) twice, with pellets resuspended in PBS. G-ELNs were stored at -80°C. Nanoparticle tracking analysis (ZetaView x30, Germany) evaluated size distribution. Protein content was quantified via BCA assay (Beyotime, China). TEM (JEM-2100Plus, Japan) imaging used phosphotungstic acid-negative staining.

**2.PKH26 Labeling and RAW264.7 Cellular Uptake Assay**

G-ELNs (50 μg) were labeled with PKH26 (Umibio, China) by vortexing (3000 rpm, 1 min) and incubating in the dark (10 min). Free dye was removed by ultracentrifugation. RAW264.7 cells (2×10^4^/well) were incubated with labeled G-ELNs (50 μg/mL, 3 h), fixed with 4% PFA, permeabilized (0.1% Triton X-100), blocked with 5% BSA, and stained with FITC-phalloidin (cytoskeleton) and DAPI (nuclei). Images were acquired via structured illumination microscopy (Nikon, Japan; 405/488/555 nm excitation).

**3.Quantitative Real-Time Polymerase Chain Reaction (qRT-PCR) for Gene Expression Analysis**

RAW264.7 cells were seeded in 6-well plates (3×10^5^ cells/well) and cultured for 24 h. RAW264.7 cells were treated with 100 μg/mL G-ELNs in DMEM for 48 h. Then, total RNA was extracted with RNA extraction kit (Promega, China) and the RNA purity and concentration were measured by Nanodrop (Thermo Scientific, USA). cDNA synthesis was performed with 1 μg RNA using the PrimeScript RT kit (TaKaRa, Japan) (37°C for 15 min, 85°C for 5 s). qRT-PCR was performed using TB Green® Premix Ex Taq™ II (TaKaRa, Japan) in a 20 μL reaction volume containing: 10 μL 2×TB Green Premix, 0.4 μL each of forward/reverse primers (10 μM), 2 μL cDNA, and 7.2 μL nuclease-free water. Thermal cycling: 95°C for 30 s (pre-denaturation), 40 cycles of 95°C for 5 s (denaturation) and 60°C for 30 s (annealing/extension), and then a melt curve analysis. GAPDH was used as the reference gene. Target genes were M1 markers (*iNOS*), M2 markers (*Il-4, Il-10, Arg-1, TGF-β*), and efferocytosis related genes. Relative expression was calculated by 2^-ΔΔCt^. The experiments were performed in triplicates.

**4.EdU Staining for RAW264.7 Cell Proliferation**

RAW264.7 cells (4×10^4^/well in 24-well plates) were exposed to G-ELNs (0, 10, 100, 1000 μg/mL) for 24 h. EdU (50 μM) was added for an additional 2 h. Thereafter, the cells were fixed with 4% PFA, permeabilized with 0.1% Triton X-100 and stained using the EdU detection kit (Beyotime, China). Finally, the nuclei were counterstained with DAPI (1 μg/mL). Finally, fluorescence images were acquired using a Nikon inverted fluorescence microscope (Japan). The number of EdU-positive cells was calculated from five random fields per sample using ImageJ software. The experiment was independently repeated three times.

**5.Polarization Phenotype Analysis of RAW264.7 Cells**

**5.1 Flow Cytometry for M1 (CD86)/M2 (CD206) Phenotypes**

RAW264.7 cells (2×10^5^/well in 6-well plates) were incubated with PBS (Con) or G-ELNs for 24 h. RAW264.7 cells were harvested with trypsin-EDTA, resuspended and centrifuged (200×g, 4℃, 5 min) and stained with PE-conjugated anti-mouse CD86 and APC-conjugated anti-mouse CD206 (BioLegend, USA). Isotype control was included. Flow cytometry (CytoFLEX, BD Biosciences, USA) was gated on Zombie-NIR⁻ live cells (FSC-A/SSC-A for debris exclusion, FSC-A/FSC-H for doublet removal). CD86⁺ (M1) percentage and CD206⁺ (M2) percentage were calculated and M2/CD86⁺ ratio was used to represent polarization (three replicates).

**5.2 Confocal Laser Scanning Microscopy for M2 Markers (CD163, CD206)**

RAW264.7 cells (5×10^4^/well in 24-well plates with coverslips) and BMDM were cultured with PBS or G-ELNs for 48 h. The cells were fixed with 4% PFA and permeabilized with 0.1% Triton X-100 in 1% BSA for 10 min and then blocked with 5% BSA for 1 h. The cells were incubated with rabbit anti-mouse CD163 or CD206 (Abcam, UK) overnight at 4°C, followed by secondary 488-conjugated goat anti-rabbit antibodies (Abcam, UK) and DAPI. Images were acquired under CLSM (Nikon, Japan; excitation, 405/488 nm). MFI of CD163⁺ or CD206⁺ cells was calculated by ImageJ (five fields/sample, three replicates).

**6. Bioinformatics Analysis**

Total RNA from RAW264.7 cells (Con vs. G-ELNs-treated groups, with biological replicates) underwent transcriptome sequencing. Raw sequencing data were quality-controlled, filtered, aligned to the reference genome, and quantified for gene expression. Differentially expressed genes (DEGs) were identified using DESeq2 (R package) based on predefined significance thresholds. Heatmaps and volcano plots were generated to visualize DEGs. Functional enrichment analyses included Gene Ontology (GO) for efferocytosis-related biological processes/molecular functions, Kyoto Encyclopedia of Genes and Genomes (KEGG) pathway analysis, and Gene Set Enrichment Analysis (GSEA) for efferocytosis-related gene sets. Protein-protein interaction networks of key efferocytosis-related DEGs (e.g., phosphatidylserine-binding receptors) were constructed using interaction databases and visualized via standard tools. All sequencing and bioinformatics analyses were conducted by Hangzhou Lianchuan Biotechnology Co., Ltd.

**7. In Vitro Efferocytosis Assays**

**7.1 Flow Cytometry for Efferocytosis Rate**

UV-irradiation (1.5 h at 254 and 290 nm) of Jurkat cells was used to induce apoptosis as revealed by Annexin V-FITC/PI staining and labeling with PKH26 (1 μM, 37°C, 20 min). RAW264.7 cells (2×10^5^/well in 6-well plates, PBS or G-ELNs for pretreatment for 24 h) were co-cultured with PKH26-labeled apoptotic Jurkat cells (1:5) for 2 h. After washing, the cells were stained with FITC anti-F4/80 (BioLegend, USA) and analyzed by flow cytometry (BD Biosciences, USA). Efferocytosis rate was calculated as the percentage of F4/80⁺ RAW264.7 cells positive for PKH26 (three replicates in total).

**7.2 Immunofluorescence-Based Efferocytosis Index**

RAW264.7 cells (3×10^3^/well in 96-well plates, G-ELNs, treated with 48 h) were co-cultured with PKH26-labeled apoptotic Jurkat cells (1:5) for 2 h. RAW264.7 cells were fixed (4% PFA), permeabilized (0.1% Triton X-100), blocked (5% BSA) and stained with FITC-phalloidin (cytoskeleton) and DAPI (nuclei). Images were acquired using a high content confocal microscope (PerkinElmer, USA). Efferocytosis index was calculated as the mean fluorescence intensity (MFI) of FITC⁺PKH26⁺ cells from at least five fields/sample (three replicates).

**8. Sequential Efferocytosis Assay via Flow Cytometry**

Two batches of UV-irradiated Jurkat cells were stained with PKH26 (Batch 1) or PKH67 (Batch 2). RAW264.7 cells (2×10^5^/well, PBS or G-ELNs-treated for 24 h) were cocultured with PKH26-stained apoptotic cells (1:5, PBS or G-ELNs-treated for 24 h) for 2 h, washed and resuspended in culture medium with PKH67-stained apoptotic cells (1:5, PBS or G-ELNs-treated for 24 h) for another 2 h. Subsequently, cells were stained with FITC-F4/80 antibody and analyzed under a flow cytometer. Sequential efferocytosis capacity was calculated as the percentage of F4/80⁺RAW264.7 cells double-stained with PKH26/PKH67.

**9. Preparation of Small Intestinal Submucosa (SIS)**

Fresh porcine jejunum segments (10 cm) were rinsed with sterile saline to remove luminal contents. The outer layers (mucosa, serosa, circular/longitudinal muscle, and adipose tissue) were mechanically stripped, retaining only the submucosa. The tissue was rinsed with sterile saline (×3), immersed in chloroform-methanol (1:1 v/v) for 12 h (room temperature, dark) to delipidize, washed with deionized water (×5, 10 min each), and treated with 0.05% trypsin-EDTA (37°C, 12 h) to remove residual cells. After saline washes (×3), the tissue was incubated in 0.5% SDS (room temperature, 4 h, 100 rpm agitation) to eliminate proteins, rinsed until clear, disinfected with 0.1% peracetic acid (30 min), and washed with deionized water (×6, 15 min each). The SIS was lyophilized (-80°C pre-freeze, 48 h freeze-drying) and stored desiccated.

**10. SIS Methacrylated Hydrogel (SM) Preparation and Characterization**

Lyophilized SIS was cryo-ground (Retsch, Germany) into powder (≤100 μm). SIS powder (300 mg) was digested in 0.1 M HCl (10 mL) containing 30 mg pepsin (25°C, 60 rpm, 72 h). Methyl methacrylate (MMA, 1:1 mass ratio to SIS) was added, pH adjusted to 7.0 with 1 M NaOH, and stirred (300 rpm, 24 h) for methacrylation. The solution was dialyzed (3.5 kDa MWCO, Sigma-Aldrich, USA) against sterile deionized water (5 days, 3× daily buffer change) and lyophilized into SM powder. For hydrogel formation, SM powder (100 mg/mL) was dissolved in PBS with 1% LAP, cast into molds, and crosslinked under 405 nm light (10 mW/cm², 10 s). Freeze-dried SM hydrogel was sputter-coated with gold (10 nm) and imaged via SEM (Zeiss, Germany) for surface morphology and porosity analysis.

**11. Biocompatibility Evaluation of SM Hydrogel**

**11.1 CCK-8 Proliferation Assay**

HUVECs and NIH-3T3 (1×10^3^/well in 96-well plates) were cultured with material extracts. At 1/3/5 days, CCK-8 reagent (Yeason, China) was added (100 μL/well), incubated (37°C, 2 h, dark), and absorbance (450 nm) measured via microplate reader (BioTek, USA).

**11.2 Hemolysis Assay**

Rabbit RBCs (Darshu Experimental Animal Co., China) were isolated (3.8% sodium citrate, 1000×g, 4°C, 10 min), washed (PBS, ×3), and resuspended as 5% (v/v) suspension. SM hydrogel (50 mg) was incubated with RBC suspension (500 μL, 37°C, 60 min). After centrifugation (1000×g, 4°C, 10 min), supernatant absorbance (540 nm) was measured. Hemolysis rate (%) = $\frac{\text{（}\text{OD value of experimental group-OD value of negative control group}\text{）}}{\text{（}\text{OD value of positive control group-OD value of negative control group}\text{）}}\text{×100\%}$，where negative control = PBS (0% hemolysis) and positive control = 0.1% Triton X-100 (100% hemolysis). Triplicates were performed per group.

**12. In Vitro Release Profile of G-ELNs from SM Hydrogel**

G-ELNs@SM hydrogel was prepared by mixing 500 μg G-ELNs with 1 mL SM solution (100 mg/mL containing 1% LAP) and crosslinking via 405 nm light irradiation (10 s). The hydrogel was incubated in 2 mL sterile PBS (pH 7.4, 0.02% sodium azide) at 37°C with 50 rpm agitation. Release medium was collected at 0, 3, 6, 9, 12, 15, 18, and 21 days, replaced with fresh PBS each time. G-ELNs protein content in the release medium was quantified using a BCA protein assay kit (Beyotime, China). Cumulative release amount and release rate (%) were calculated, and release curves were plotted from triplicate experiments.

**13. Diabetic Full-Thickness Skin Defect Model and Wound Treatment**

Eighty male SD rats (8 weeks old) were acclimatized for 1 week. Diabetes was induced via intraperitoneal injection of streptozotocin (STZ, Sigma-Aldrich, USA; 50 mg/kg in 0.1 M citrate buffer, pH 4.5). Rats with fasting blood glucose ≥16.67 mmol/L after 1 week (n=64) were selected, with 16 non-diabetic rats as controls. Under isoflurane anesthesia (5% induction, 2% maintenance), four 10-mm full-thickness dorsal skin defects (down to fascia) were created. Wounds were treated as follows: 1) Saline (500 μL); 2) Pure SM hydrogel (500 μL); 3) Free G-ELNs (50 μg in 500 μL saline); 4) SM@G-ELNs hydrogel (50 μg G-ELNs in 500 μL hydrogel). Each group (5 total: 4 diabetic + 1 non-diabetic control) contained 4 rats. Wounds were dressed with sterile gauze and secured with Vaseline tape. Wound healing was monitored daily; digital images were captured at 0/3/7/14/21 days post-surgery. Remaining wound area was measured via ImageJ, with healing rate (%) = $\frac{\text{（}\text{Initial area-Residual area}\text{）}}{Initial area}\text{×100\%}$

**14. Histological and Immunohistochemical Analysis of Wound Tissue**

All rats were euthanized with pentobarbital sodium (100 mg/kg) at 3, 7, 21 days after surgery. Full-thickness wound tissues (10 mm including the margin of wound) were fixed in 10% neutral buffered formalin (24h), dehydrated in graded alcohol, embedded in paraffin, and sectioned(3μm). (1) H&E staining：Stained with hematoxylin (5 min), differentiated in 1% acid ethanol (30 s), stained with eosin (3 min) and then observed the inflammation and granulation tissue. (2) Masson’s trichrome: Stained with Weigert’s iron hematoxylin (5 min), ponceau-acid fuchsin (10 min), phosphomolybdic acid (5 min), and aniline blue (5 min) and then observed the collagen deposit. (3) Sirius red staining: Stained with picrosirius red (1 h), rinse and observe under polarized light (type I collagen: red; type III: green). (4) IHC staining: After endogenous peroxidase blocking(3%H₂O₂), antigen retrieval (citrate buffer, pH 6.0), stained with primary antibodies: anti-CD163, anti-CD68, anti-Caspase-3, anti-MERTK (Abcam, UK; 4℃, overnight), and then incubated with secondary antibodies Alexa Fluor 488/594 conjugated with(1h), and then incubated with DAB chromogen, and then nuclei were stained with hematoxylin. Positive cells or stained area were counted in six fields/section based on the Image J.

**15. Establishment of Jurkat Cell Apoptosis Model**

The Jurkat cell apoptosis model was induced by UVC irradiation (wavelength 254 nm), with irradiation doses optimized through gradient pre-experiments. Post-treatment cells were dual-stained with Annexin V-FITC and propidium iodide (PI), and fluorescence signals were acquired using a flow cytometer (e.g., BD FACSCanto II) to distinguish early apoptotic (Annexin V⁺/PI⁻) and late apoptotic/necrotic (Annexin V⁺/PI⁺) populations. Each experimental group included three independent biological replicates. Data were analyzed via FlowJo software to calculate apoptosis percentages and standard deviations. The model was deemed successful when the total apoptosis rate (early + late apoptosis) reached ≥80%, and a dynamic apoptosis model was constructed based on time gradients (0 ~ 1.5 hours) meeting this threshold.

**16. TUNEL Staining for Apoptosis Detection in Skin Tissues**

**Paraffin-embedded skin tissue sections underwent sequential dewaxing in xylene I, Ⅱ, and Ⅲ (10 minutes each), followed by rehydration through graded ethanol (100%, 95%, 80%, 5 minutes per gradient). Protease K (20 μg/mL in pH 7.4 Tris-HCl buffer) was applied at 37°C for 15 minutes to expose DNA breaks. After PBS rinsing, TUNEL reaction mixture (containing fluorescein-dUTP and TdT enzyme) was added and incubated at 37°C for 1 hour in the dark, with negative control slides lacking TdT enzyme to exclude nonspecific staining. Nuclei were counterstained with DAPI (1 μg/mL) for 5 minutes in the dark. A laser confocal microscope (excitation wavelengths: TUNEL 488 nm, DAPI 358 nm) captured Z-stack images under single-exposure conditions (≤500 ms) to avoid fluorescence quenching. Specificity was validated by confirming green fluorescence signals exclusively in experimental groups.**

**17. Single-Cell Normalized Mean Fluorescence Intensity Quantification**

**Mean fluorescence intensity (MFI) quantification was performed using ImageJ 1.53. Fluorescence channels were split to isolate target protein and nuclear (DAPI) signals. Nuclear segmentation was achieved via threshold adjustmentto generate a binary mask, which was applied to the target protein channel for measuring integrated fluorescence intensity within each nuclear region. Single-cell corrected MFI was calculated by normalizing the target protein's integrated intensity against either nuclear area or DAPI integrated intensity. Whole-section MFI was derived from the mean of all single-cell corrected MFI values.**

**18. Western Blot Analysis**

**(1) Western Blot Analysis of RAW264.7 Cells**

RAW264.7 macrophages were seeded in 6-well plates and treated as indicated. After treatment, cells were washed twice with ice-cold PBS and lysed in RIPA buffer supplemented with protease and phosphatase inhibitor cocktails on ice for 30 min. Cell lysates were centrifuged at 12,000 × g for 15 min at 4°C, and the supernatants were collected. Equal amounts of protein (20 μg) were separated by 10% SDS-PAGE and transferred onto PVDF membranes (Millipore, USA). The membranes were blocked with 5% skim milk in TBST for 1 h at room temperature, followed by overnight incubation at 4°C with primary antibodies against MERTK (1:1000, Proteintech, China), ARG1 (1:2000, Abcam, UK), and β-actin (1:100,000, Abclonal, China) as a loading control. After washing three times with TBST, the membranes were incubated with HRP-conjugated secondary antibodies (1:5000, Abclonal, China) for 1 h at room temperature. Protein bands were visualized using an enhanced chemiluminescence (ECL) detection system.

**(2) Western Blot Analysis of G-ELNs**

Purified G-ELNs were lysed in RIPA buffer containing protease inhibitors on ice for 30 min. Equal amounts of G-ELN protein were mixed with 5× loading buffer, heated at 95°C for 10 min, and then separated by 10–12% SDS-PAGE. Proteins were transferred onto PVDF membranes, which were then blocked with 5% skim milk in TBST for 1 h at room temperature. The membranes were incubated overnight at 4°C with primary antibodies against exosomal markers, including HSP70 and TET8, as well as the negative control β-actin. After washing, the membranes were incubated with HRP-conjugated secondary antibodies for 1 h at room temperature. Protein bands were visualized using an ECL detection system.

**(3) Western Blot Analysis of Wound Tissues**

**Wound tissue samples were harvested from each group at day 14 post-treatment and immediately frozen in liquid nitrogen. Tissues were homogenized in ice-cold RIPA buffer supplemented with protease and phosphatase inhibitors using a tissue grinder. The homogenates were incubated on ice for 30 min and then centrifuged at 12,000 × g for 15 min at 4°C to remove debris. Equal amounts of protein (20 μg) were separated by 10% SDS-PAGE and transferred onto PVDF membranes. Membranes were blocked with 5% skim milk in TBST for 1 h at room temperature and then incubated overnight at 4°C with primary antibodies against MERTK (1:1000, Proteintech, China) and β-actin (1:100,000, Abclonal, China), which served as a loading control. After washing three times with TBST, membranes were incubated with HRP-conjugated secondary antibodies (1:5000, Abclonal, China) for 1 h at room temperature. Protein bands were visualized using an enhanced chemiluminescence (ECL) detection system**

1. **Immunofluorescence Staining for Collagen Types I and III in Rat Skin Tissue**

**Rat skin tissues were fixed in 4% paraformaldehyde for 24 hours, embedded in OCT compound, and sectioned at 5-μm thickness. After antigen retrieval with citrate buffer (pH 6.0) and blocking with 5% goat serum, sections were incubated overnight at 4°C with primary antibodies: anti-COL1A1 (72026, Cell Signaling Technology) and anti-COL3A1 (NBP1-05119, Novus Biologicals). Following PBS washes, sections were incubated with secondary antibodies: goat anti-rabbit IgG-Alexa Fluor 488 (ab150077, Abcam) and donkey anti-mouse IgG-Alexa Fluor 594 (115-025-003, Jackson ImmunoResearch) for 1 hour at room temperature. Nuclei were counterstained with DAPI, and sections were mounted with antifade medium. Images were captured using a confocal microscope.**

1. **Metabolomics Analysis of G-ELNs**

**The metabolomic profiling of grape-derived exosome-like nanoparticles (G-ELNs) was conducted by Novogene Co., Ltd (Beijing, China), Ltd using their standardized quasi-targeted platform. Lyophilized samples underwent methanol/water extraction and were analyzed via LC-MS/MS. Metabolite identification utilized Novogene's proprietary database with MRM mode, and statistical validation employed multivariate analysis to confirm key metabolites.**

**Table S1. The primer sequences used in qRT-qPCR analysis.**

***Mus musculus***

| Gene | Forward (5'→3') | Reverse (5'→3') |
| --- | --- | --- |
| *Gapdh* | TGCACCACCAACTGCTTAGC | GGCATGGACTGTGGTCATGAG |
| *Il-4* | CCCCAGCTAGTTGTCATCCTG | CAAGTGATTTTTGTCGCATCCG |
| *Il-10* | CCCATTCCTCGTCACGATCTC | TCAGACTGGTTTGGGATAGGTTT |
| *Arg-1* | GGAATCTGCATGGGCAACCTGTGT | AGGGTCTACGTCTCGCAAGCCA |
| *Tgf-β* | CTCCAAGCCAAAGTCCTTAGAG | AGGAGCTGTCATTAGGGACATC |
| *iNOS* | TCTTGGAGCGAGTTGTGGATTGT | TAGGTGAGGGCTTGCCTGAGTG |
| *Mertk* | CAGGGCCTTTACCAGGGAGA | TGTGTGCTGGATGTGATCTTC |
| *Axl* | GGAACCCAGGGAATATCACAGG | AGTTCTAGGATCTGTCCATCTCG |
| *Tyro3* | AGTATGGCTCACTGTCGAAGG | AGTAAATGGTTACGGGTTCTGGA |
| *Gas6* | GAACTTGCCAGGCTCCTACTCT | GGAGTTGACACAGGTCTGCTCA |
| *Rac1* | GTCCGTGCAAAGTGGTATCCT | GCACCGATCTCTTTCGCCAT |

***Rattus norvegicus***

| Gene | Forward (5'→3') | Reverse (5'→3') |
| --- | --- | --- |
| *Gapdh* | CAAGTTCAACGGCACAGTCA | CACCCCATTTGATGTTAGCG |
| *Il-4* | CGTGATGTACCTCCGTGCTT | GTGAGTTCAGACCGCTGACA |
| *Il-10* | GCTCAGCACTGCTATGTTGC | TTGTCACCCCGGATGGAATG |
| *Arg-1* | TAAGGAAAGCTGGCCTGGTG | GCTGTCATTGGGGACATCCA |
| *Tgf-β* | GACCGCAACAACGCAATCTA | CGTGTTGCTCCACAGTTGAC |
| *iNOS* | ACAAGCTGCATGTGACTCCATC | GTCCTCTGGTCAAACTCTTGGAG |
| *Mertk* | CGCAGGGCTAGACATGAACA | TCTTCCCATTCCTCGTTGCC |
| *Axl* | ACGTCAACATGGATGAGGGC | GACATAGCGTCCAGCCGAAT |
| *Tyro3* | CTTCACATGCCTGCGAATGG | CCAGGGCTGAAGATGTACCG |
| *Gas6* | AAGTGGATGGTACCAAGGGC | CCACAAAGGTGAGCACGGA |
| *Rac1* | CTAGTGGGGACGAAGCTTGA | CAGAGAACGGCTCGGATAGC |


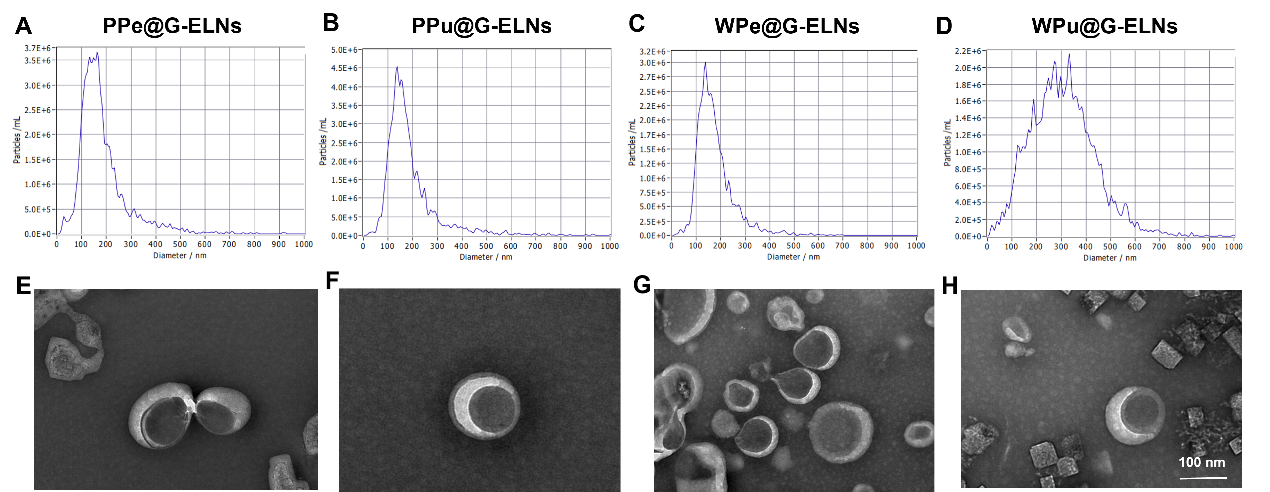


**Figure S1. Characterization of White and Purple Grape Exosome-like Nanovesicles.** (A-D) Nanoparticle tracking analysis of size distribution for purple grape peel-derived exosome-like nanovesicles (PPe@G-ELNs), purple grape pulp-derived exosome-like nanovesicles (PPu@G-ELNs), white grape peel-derived exosome-like nanovesicles (WPe@G-ELNs), and white grape pulp-derived exosome-like nanovesicles (WPu@G-ELNs). (E-H) Transmission electron microscopy images of PPe@G-ELNs, PPu@G-ELNs, WPe@G-ELNs, and WPu@G-ELNs.


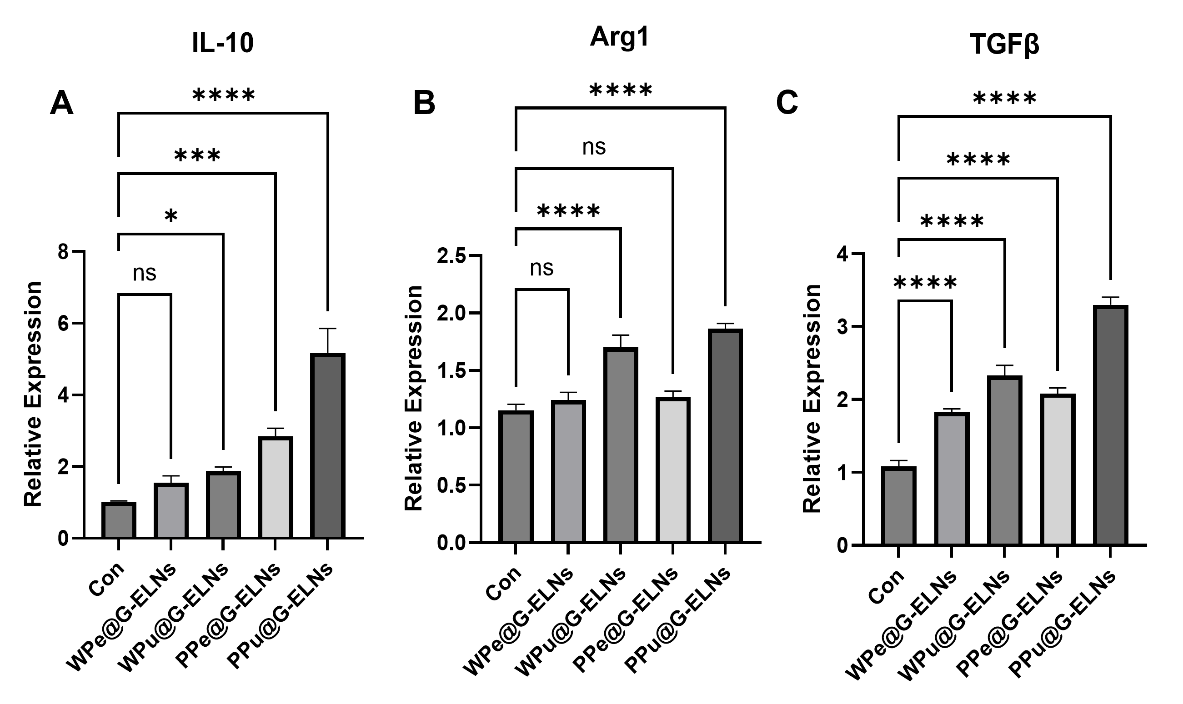


**Figure S2. mRNA Expression Levels of Immunomodulatory Markers in RAW264.7 Cells.** (A-C) Quantitative analysis of IL-10, Arg-1, and TGF-β expression levels in RAW264.7 cells following various treatments: control group (Con), PPe@G-ELNs, PPu@G-ELNs, WPe@G-ELNs and WPu@G-ELNs.


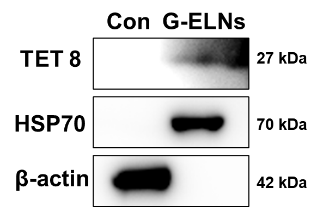


**Figure S3. Characterization of G-ELN purity by Western Blot analysis.**


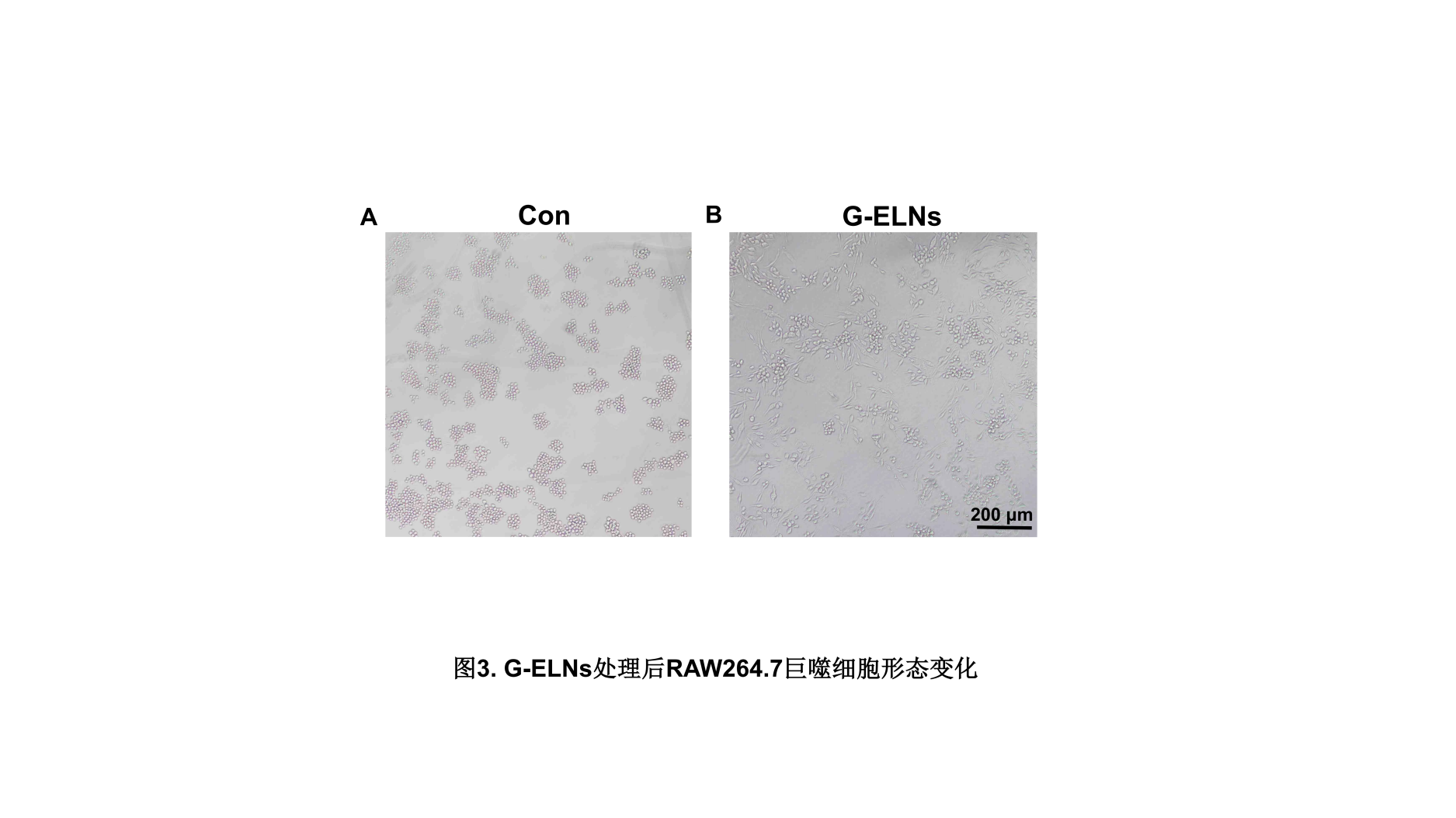


**Figure S4. Morphological Changes of RAW 264.7 Macrophages After 2-Day Treatment with Grape Exosome-Like Nanoparticles (G-ELNs)** (A) Morphology under normal conditions; (B) Morphology following G-ELNs treatment.


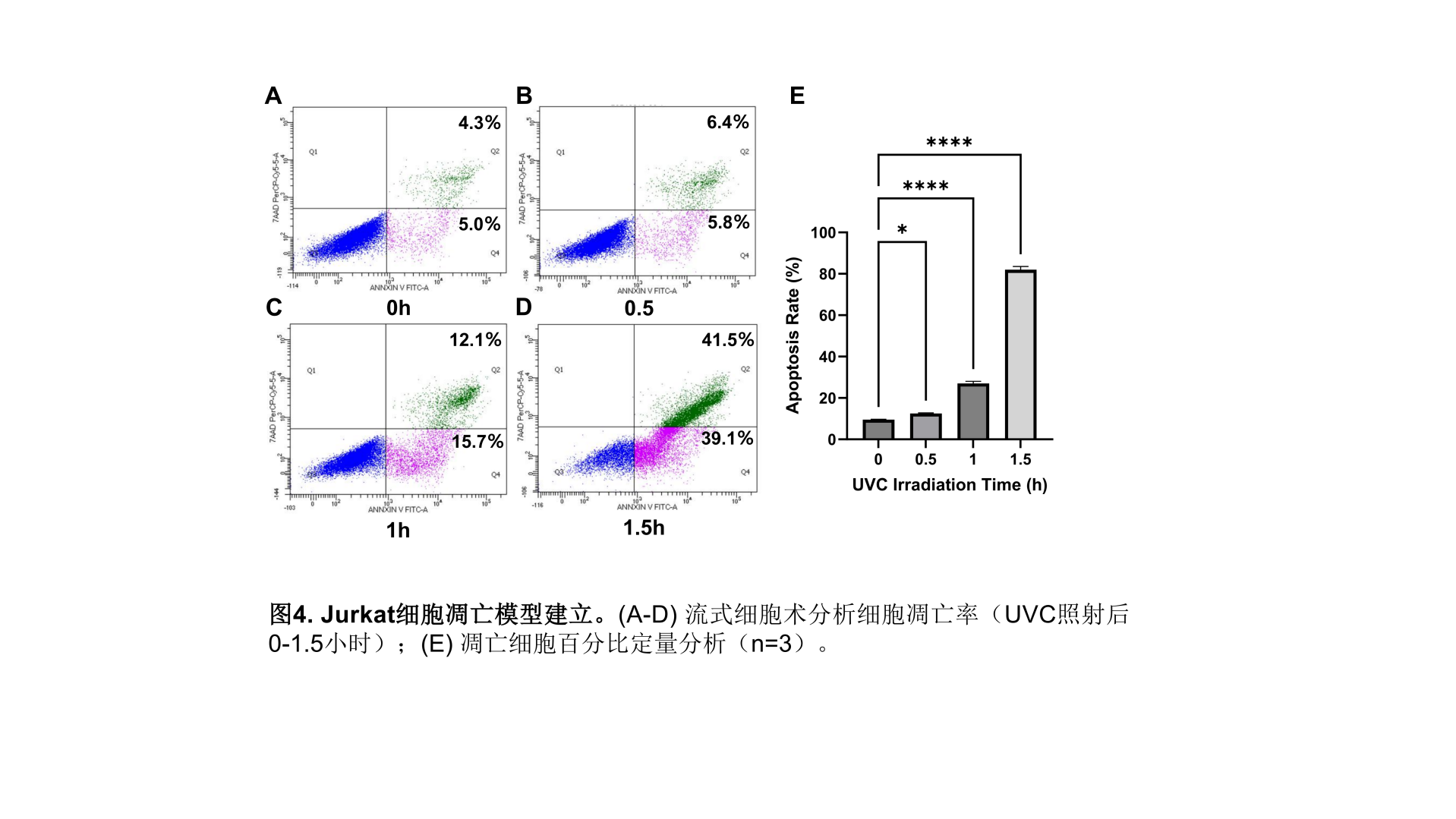


**Figure S5. Establishment of Jurkat Cell Apoptosis Model.** (A-D) Flow cytometry analysis of apoptosis rates at 0–1.5 hours post-UVC irradiation; (E) Quantitative analysis of apoptotic cell percentages (n=3).


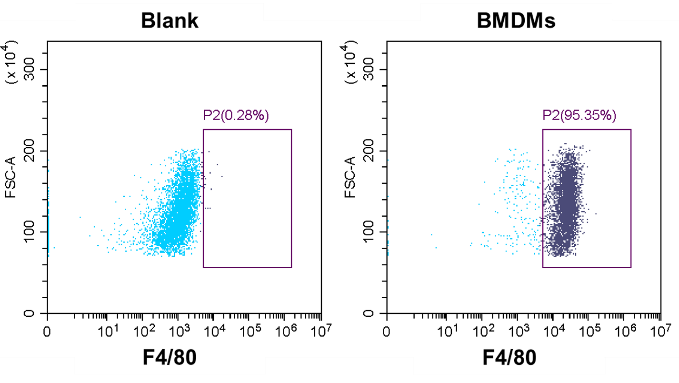


**Figure S6. Flow cytometry identification diagram of primary BMDMs**


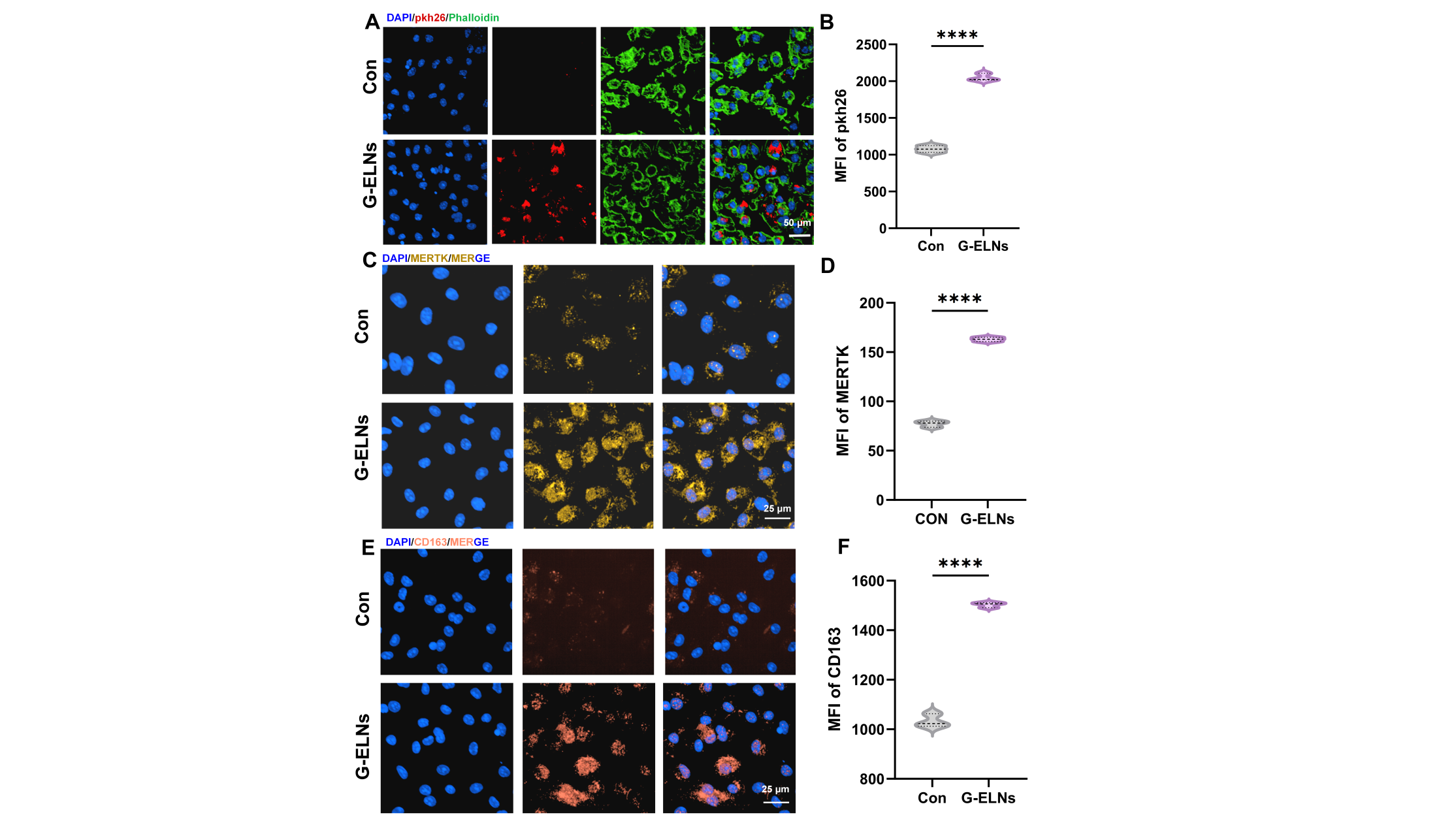


**Figure S7. G-ELNs promoted M2 phenotype polarization and efferocytosis of BMDM in vitro.** A, B) Confocal images and quantification of M2 phenotype (CD163) of RAW264.7 treated with G-ELNs. C, D) Confocal images and quantitative analysis of RAW264.7 efferocytosis after G-ELN treatment. E, F) Confocal images and quantitative analysis of MERTK protein expression in RAW264.7 treated with G-ELN. Data are presented as mean ± SD. **p < 0.1，**p < 0.01，***p < 0.001，****p < 0.0001*，ns = there was no significant difference.

**Figure S8. Cytotoxicity of UNC2025 in RAW264.7 assessed by CCK-8 assay.**


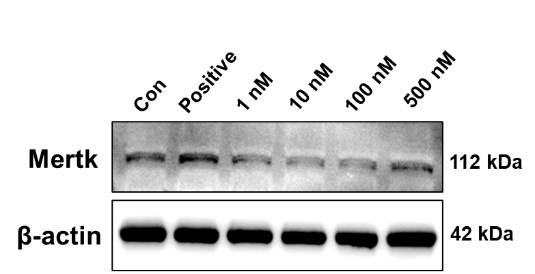


**Figure S9. MERTK protein expression in macrophages treated with increasing concentrations of UNC2025.**


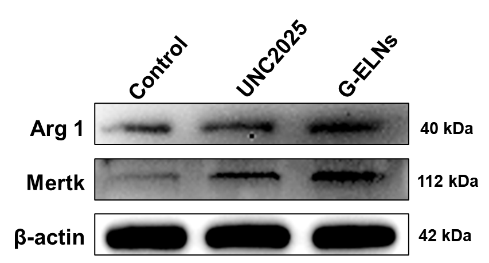
**Figure S10. MERTK functional blockade attenuates G-ELNs-induced upregulation of MERTK and ARG1 expression**

**
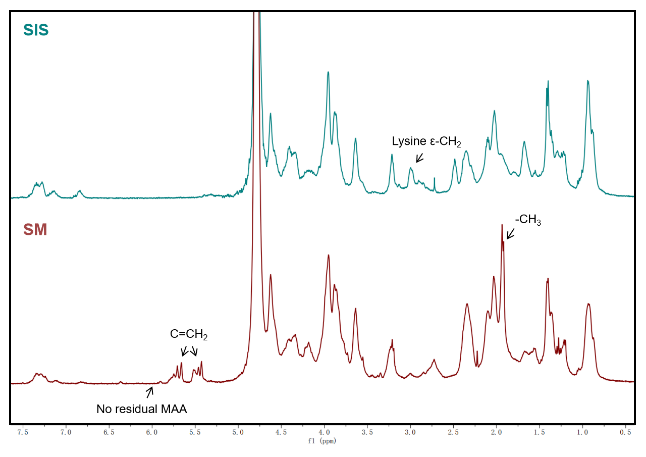
**

**Figure S11. 1H-NMR spectra of SIS and SISMA.** The successful grafting of methacryloyl groups onto the SIS backbone is confirmed by the appearance of methacrylate vinyl protons (C=CH2) at δ 5.4 and 5.7 ppm, and the methyl group (-CH3) peak at δ 1.9 ppm in the SISMA spectrum. The reduction of the lysine methylene peak (Lysine ε-CH2) at δ 2.9 ppm indicates the consumption of amino groups during the reaction. The absence of sharp peaks around 6.0 ppm (No residual MAA) confirms the thorough removal of unreacted methacrylic acid/anhydride small molecules.

**
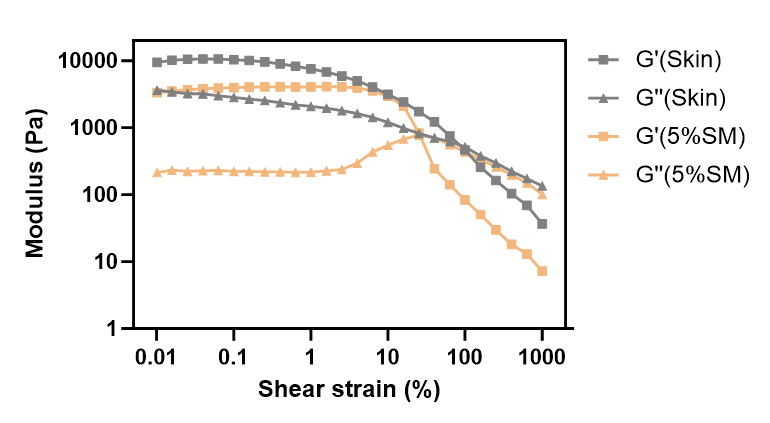
**

**Figure S12. Comparative mechanical evaluation using LAOS rheometry.** The storage (G') and loss (G'') moduli of native skin and 5% SM hydrogel are plotted against shear strain (%), demonstrating the matching viscoelastic properties and the conformable nature of the hydrogel.

**
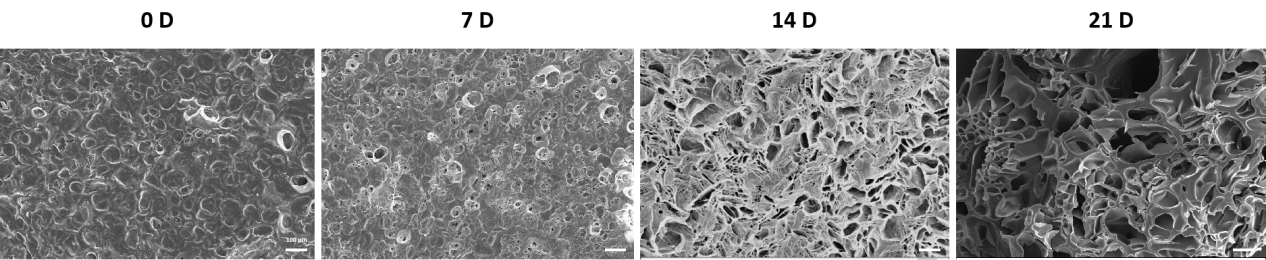
**

**Figure S13.** In vitro morphological degradation of the hydrogel construct. Representative SEM images showing the microstructural evolution and progressive pore degradation of the SM hydrogel over a 21-day period (Day 0, 7, 14, and 21). Scale bars = 100 μm.


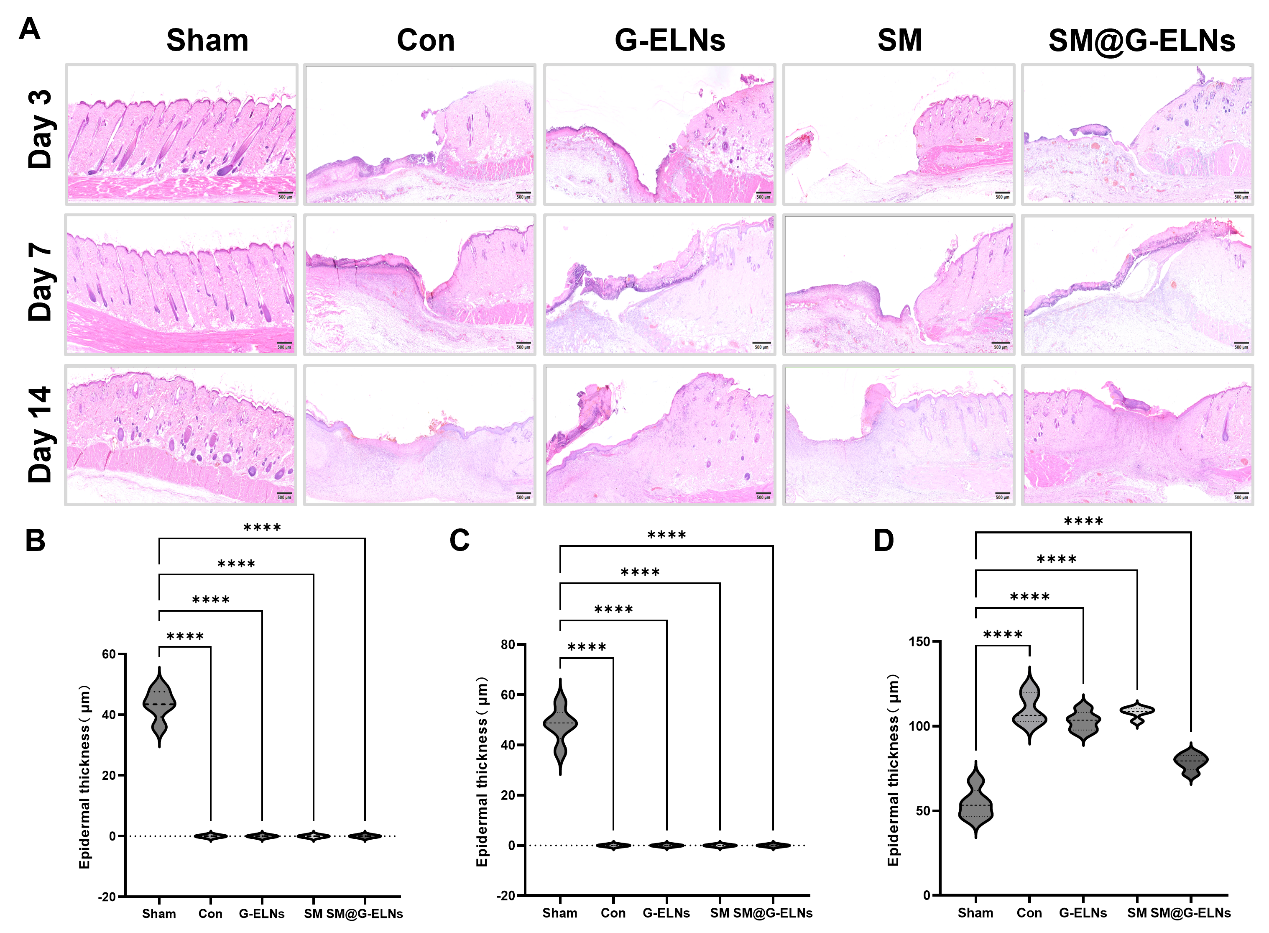


**Figure S14. Histopathological Evaluation by H&E Staining.** (A) Representative H&E-stained histological images of wound healing at days 3, 7, and 14. (B-D) Quantitative analysis of epidermal thickness during wound healing at days 3, 7, and 14.


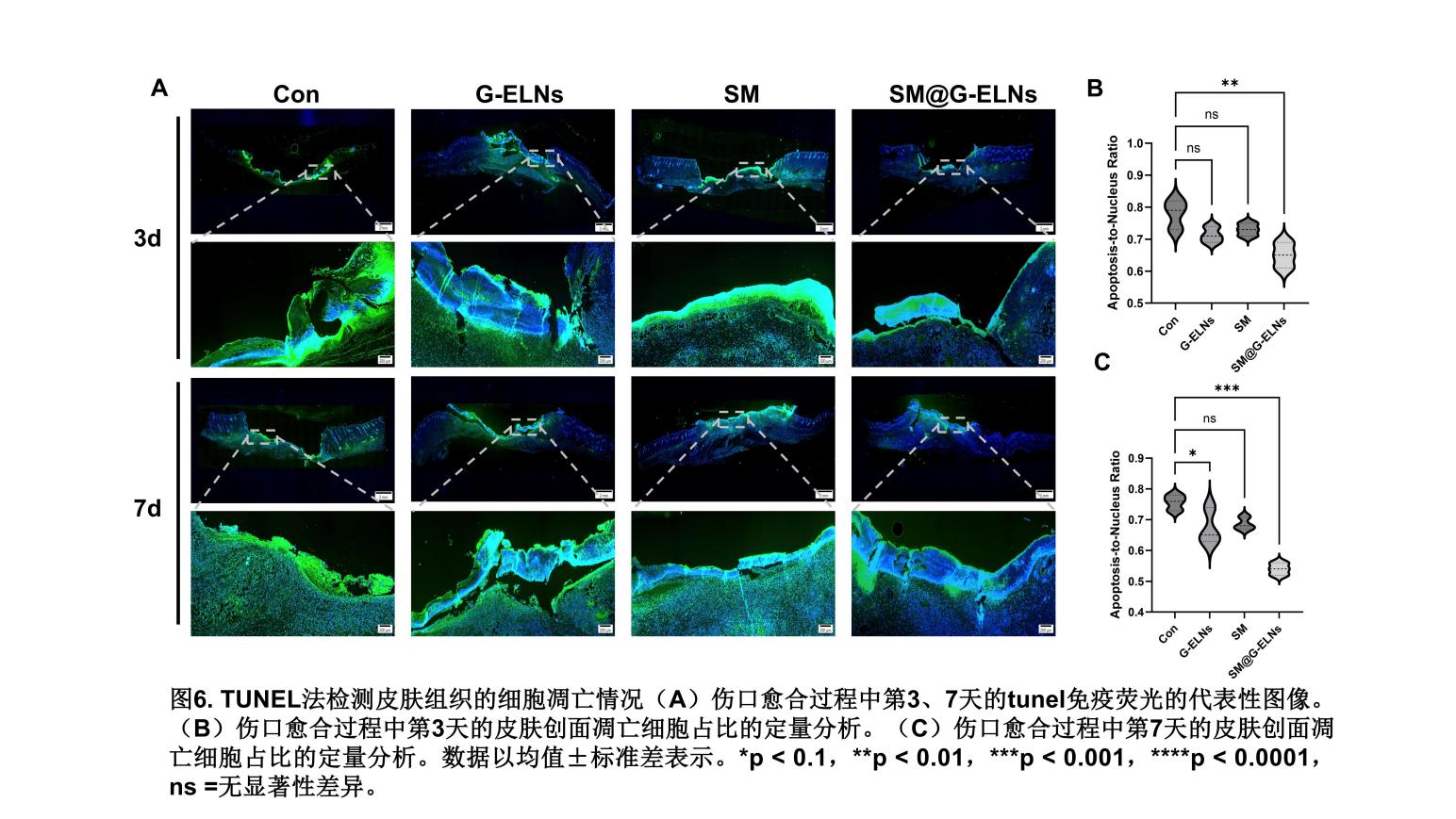


**Figure S15. Detection of Apoptotic Cells in Skin Tissues by TUNEL Assay.** (A) Representative TUNEL immunofluorescence images of skin wounds at days 3 and 7 post-injury. (B) Quantitative analysis of apoptotic cell percentage in skin wounds at day 3. (C) Quantitative analysis of apoptotic cell percentage in skin wounds at day 7.


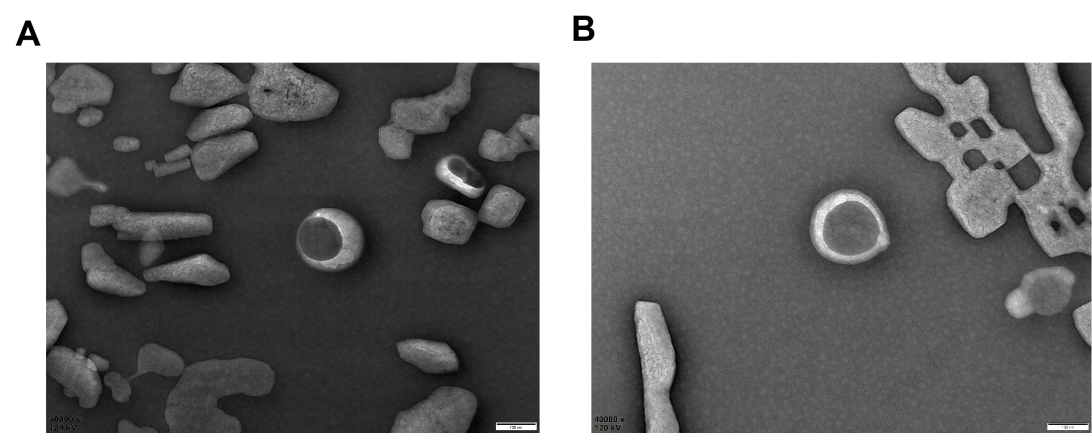


**Figure S16. Characterization of purple grape exosomes-like nanoparticles.** (A) Transmission electron microscopy image of G-ELNs before UV irradiation. (B) Transmission electron microscopy image of G-ELNs after UV irradiation.


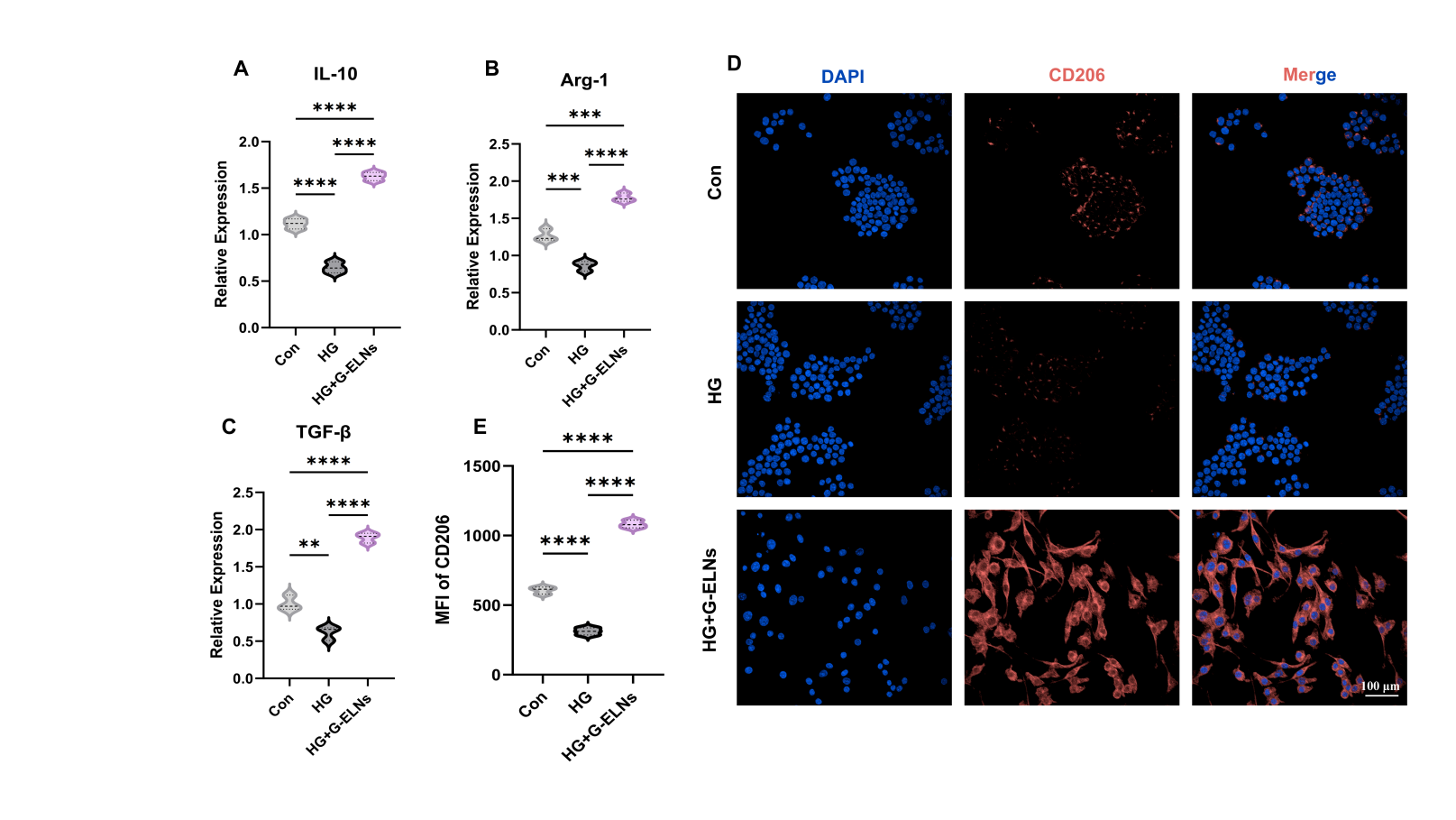


**Figure S17. G-ELNs promoted the polarization of RAW264.7 to M2 under high glucose conditions in vitro.** A-C) quantitatively analyzed the mRNA expression of IL-10, Arg-1 and TGF-β in RAW264.7 cells of different groups. D, E) Confocal images and quantitative analysis of M2 phenotype (CD206) of RAW264.7 under different treatments. Data are presented as mean±SD. **p < 0.1，**p < 0.01，***p < 0.001，****p < 0.0001*, ns = there was no significant difference.


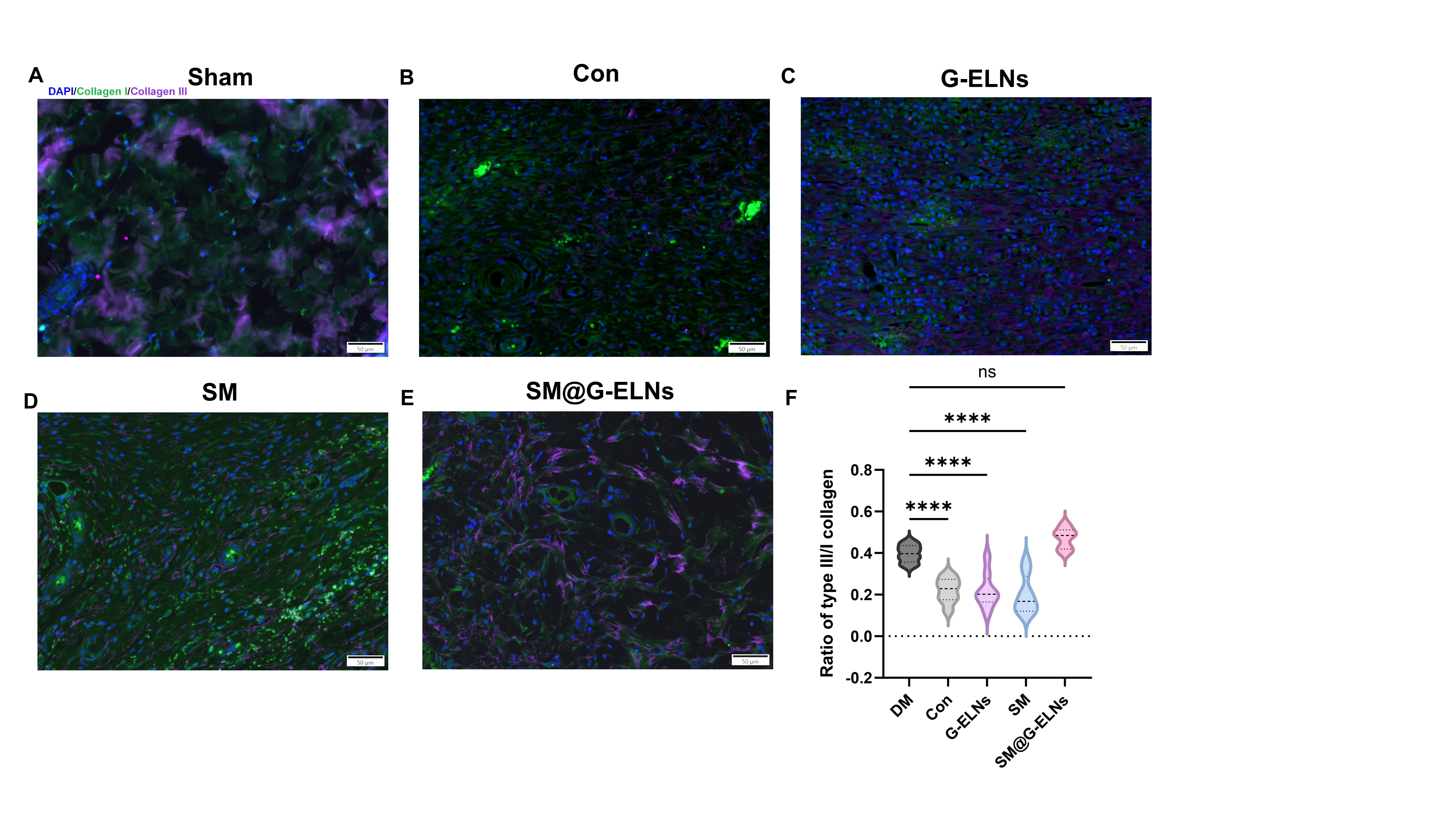


**Figure S18. Spatial distribution and quantitative analysis of type III and type I collagen during diabetic wound healing.** A-E) Confocal images of type III and type I collagen from different groups. F) Quantitative analysis of type III/I collagen ratio in different groups. *ns:p*$\text{>}$*0.05*; **p < 0.1，**p < 0.01，***p < 0.001，****p < 0.0001*.


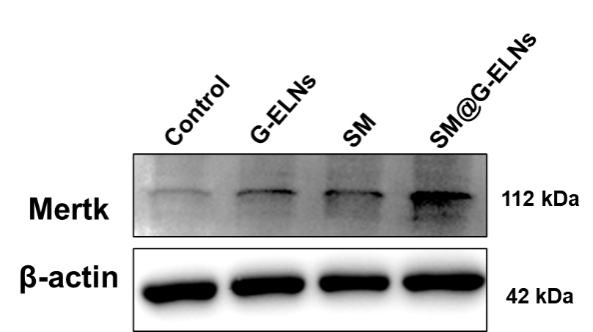
**Figure S19. Western blot analysis of MERTK protein expression in wound tissues at day 14 post-treatment.**

**Table S2. Top 10 Metabolites in G-ELNs by Relative Quantification**

| **Name** | **Formula** | **Class I** | **Relative quantitative information** |
| --- | --- | --- | --- |
| L-Malate | C_4_H_6_O_5_ | Organic acids and derivatives | 70882558948 |
| Tartaric acid | C_4_H_6_O_6_ | Organic oxygen compounds | 45310151337 |
| Citric Acid | C_6_H_8_O_7_ | Organic acids and derivatives | 32389455518 |
| Fumaric Acid | C_4_H_4_O_4_ | Organic acids and derivatives | 12868579441 |
| 5-Methoxypsoralen | C_12_H_8_O_4_ | Phenylpropanoids and polyketides | 8966764967 |
| (R)-5-Oxopyrrolidine-2-carboxylic acid | C_5_H_7_NO_3_ | Organic acids and derivatives | 2548910976 |
| N-Acetylneuraminic acid | C_11_H_19_NO_9_ | Organic oxygen compounds | 2372573948 |
| Itaconic acid | C_5_H_6_O_4_ | Lipids and lipid-like molecules | 2032761079 |
| D-Glucuronic acid | C_6_H_10_O_7_ | Organic oxygen compounds | 1666197340 |
| L-Glutamic acid | C_5_H_9_NO_4_ | Organic acids and derivatives | 1257241529 |

**Raw data of Western Blot.**

**Fig S3**

**
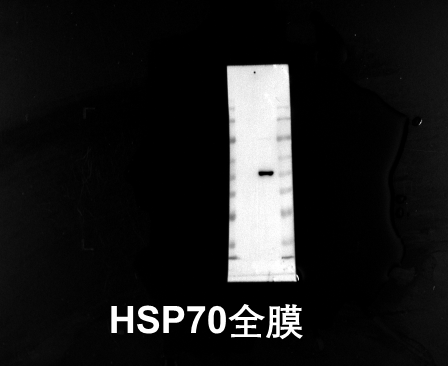
**

**
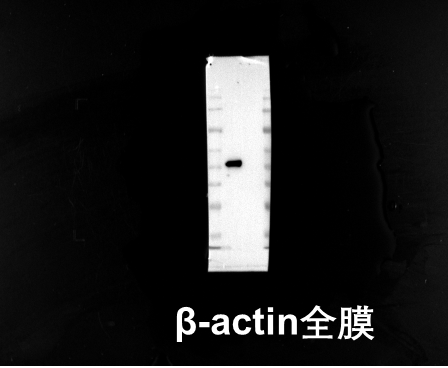
**

**
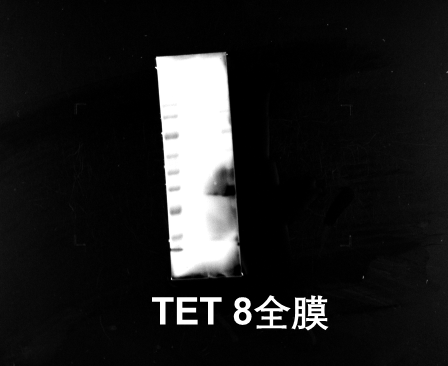
**

**Fig S9**

**
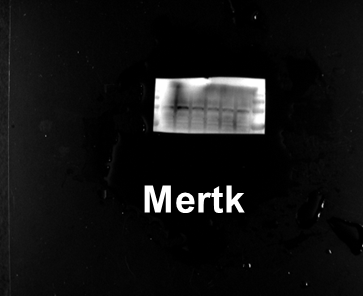
**

**
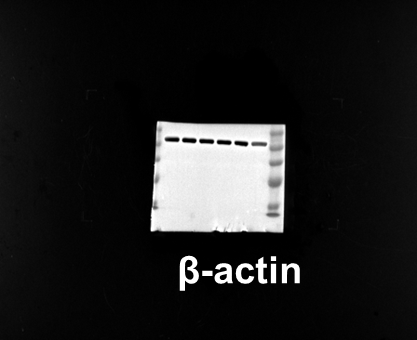
**

**
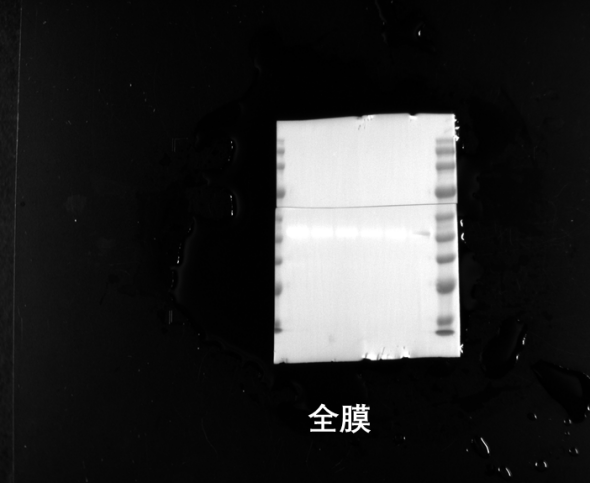
**

**Fig S10**

**
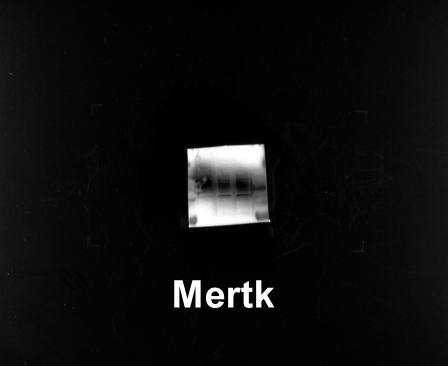
**

**
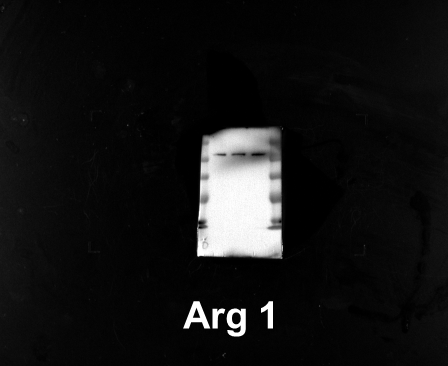
**

**
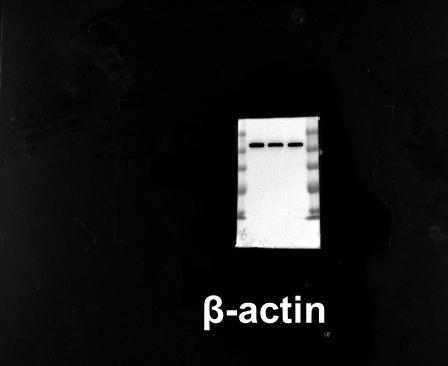
**

**
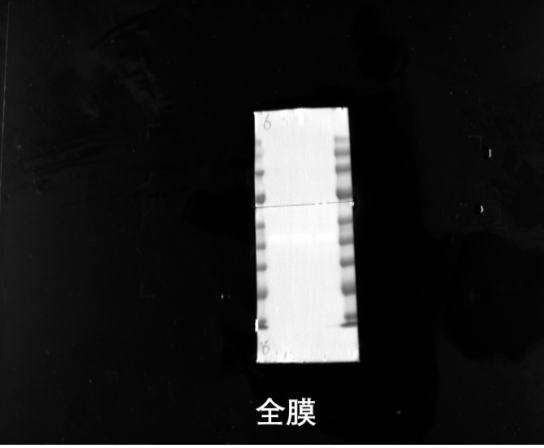
**

**Fig S19**

**
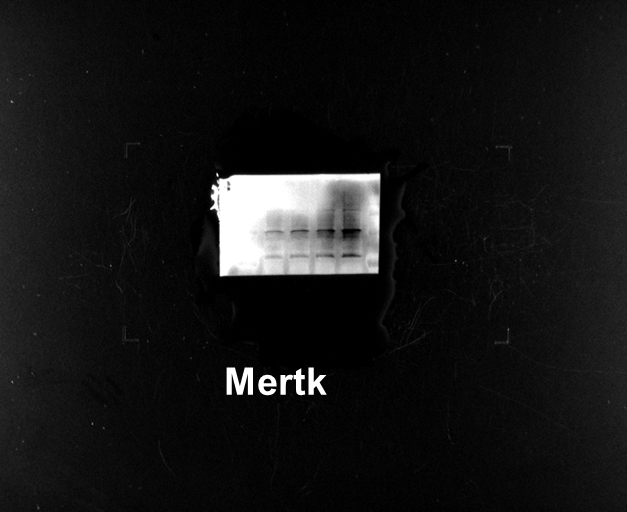
**

**
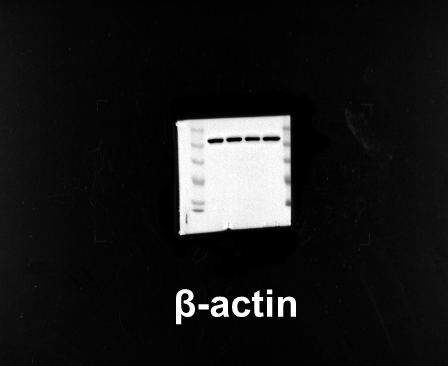
**

**
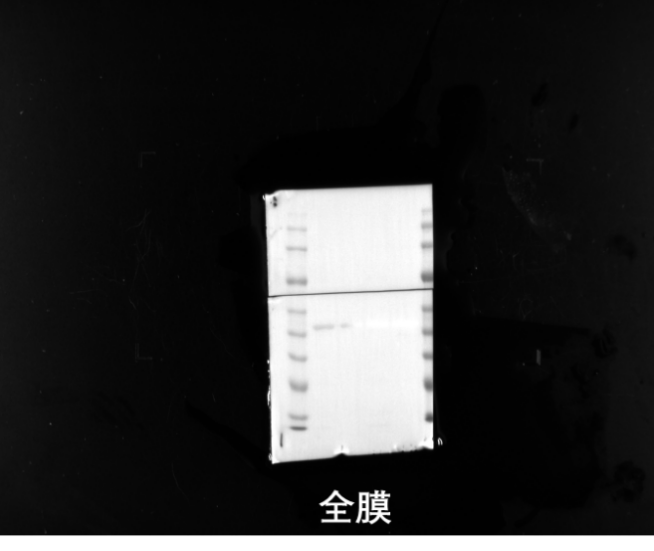
**
